# Genetic variants in *NHEJ1* and related DNA repair disorders: insights into phenotypic heterogeneity and links to hypoplastic myelodysplastic syndromes and familial hematological malignancies susceptibility

**DOI:** 10.1007/s00277-025-06257-6

**Published:** 2025-03-06

**Authors:** Mahmoud I. Elbadry, Elsayed Abdelkreem, Ahmed Tawfeek, Go Hun Seo, Shereen Philip Aziz

**Affiliations:** 1 Department of Internal Medicine, Division of Haematology, Faculty of Medicine, Sohag University, Sohag, 82524 Egypt; 2 Department of Paediatrics, Faculty of Medicine, Sohag University, Sohag, Egypt; 3 Department of Clinical and Chemical Pathology, Faculty of Medicine, Sohag University, Sohag, 82524 Egypt; 4 Medical Genetics Division, 3billion Inc, Seoul, South Korea

**Keywords:** DNA double-strand break repair, *NHEJ1* mutation – MDS, Leukaemia—Lymphoma

## Abstract

This study investigates the burden, phenotypes, progression, and outcomes of familial hematological malignancies (FHM) through clinical evaluation, gene panel testing, and whole exome sequencing, highlighting the significance of identifying genetic causes for personalized treatment. Over six years, 357 patients initially diagnosed with bone marrow failure (BMF) were evaluated, with 152 patients lacking identifiable causes undergoing further analysis. Among these, 53 (34.9%) exhibited features of inherited BMF syndromes, and 13 (24.5%) developed FHM. In a separate cohort of 27 patients with inherited immunodeficiency disorders, 8 (29.6%) developed FHM associated with *NHEJ1* or *LYST* variants, underscoring the familial clustering of hematologic disorders. Notably, 6 of 7 patients from the same family (family-1) with homozygous *NHEJ1* variants progressed to secondary myelodysplastic syndrome (sMDS), acute myeloid leukemia (AML), or lymphoma. Among 780 patients diagnosed with hematological malignancies during the study period, 45 (5.8%) were confirmed to have FHM, with 33 patients enrolled for detailed analysis. Of these, 16 (48.5%) had DNA-repair deficiencies (DNA-RD), including eight with Fanconi anemia, six with *NHEJ1* variants, and two with *BRCA2* mutations. The remaining 17 patients presented conditions such as familial myeloproliferative neoplasms, dyskeratosis congenita (DC) [*TERT*, *DKC1* variants], and Chediak-Higashi syndrome. Two siblings (family-3) with a rare *TERT* variant and a unique DC phenotype developed sMDS after prolonged BMF. Patients with DNA-RD were younger and exhibited higher rates of growth failure, recurrent infections, and endocrinopathies. These cases frequently progressed to sMDS or AML. A comparative analysis of 319 individuals with DNA double-strand break repair deficiencies revealed a 45% frequency of hematological malignancies. Lymphoma was most common in Nijmegen breakage syndrome (79.4%) while MDS/AML was prevalent in Cernunnos deficiency (66.6%). The findings emphasize the importance of early diagnosis, genetic testing, and personalized management, including timely transplantation, to improve outcomes in FHM. This research underscores the need for clinical awareness and surveillance to facilitate timely interventions and mitigate disease progression.

**Supplementary Information:**

The online version contains supplementary material available at 10.1007/s00277-025-06257-6.

## Introduction

Familial hematological malignancies (FHM) refer to a hereditary predisposition or clustering of blood-related cancers within families, leading to an increased risk of lymphomas, leukemias, myelodysplastic syndromes (MDS), and myeloproliferative neoplasms (MPNs) among multiple family members [1, 2]. These malignancies may manifest as primary clinical features or co-occur with other hematologic or systemic conditions, such as Down syndrome [3]. The diagnosis of FHM relies on identifying underlying genetic abnormalities in germline tissues [4]. Several genes, including tumor protein p53 (*TP53*), mismatch repair genes, breast cancer genes (*BRCA1*, and *BRCA2*),and ataxia-telangiectasia mutated gene (*ATM*), have been implicated in the development of FHM and are associated with an increased susceptibility to various cancers [5–7].

FHM can also be linked to specific inherited bone marrow failure syndromes (IBMFSs), such as Fanconi anemia (FA) [7], dyskeratosis congenita (DC) [8], and DNA double-strand break repair (DSBR) disorders, including Nijmegen breakage syndrome (NBS), severe combined immunodeficiency, and Ligase IV syndrome (LIG4) [9, 10]. DSBR disorders involve deficiencies in repairing double-strand breaks through pathways like Homologous Recombination (HR) and Non-Homologous End Joining (NHEJ) [11]. Variants in HR genes, such as *BRCA1* and *BRCA2* are known to increase cancer risk, particularly for breast and ovarian cancers [11]. NHEJ, also known as Cernunnos, is a rapid but error-prone repair mechanism. Variants in *NHEJ*-related genes can impair this repair process, leading to increased genomic instability and heightened cancer susceptibility [10, 12].

On the other hand, studies suggest that approximately 7% to 8% of patients initially diagnosed with sporadic MPNs are part of familial clusters, with at least two affected relatives, classifying them as familial MPNs[13–15]. In these cases, the somatic mutation in the MPN driver gene is likely acquired due to an underlying genetic predisposition. Although this predisposition has been molecularly characterized in only a limited number of families to date [15, 16], identifying familial MPN cases can facilitate early detection and diagnosis of these myeloid malignancies in at-risk family members.

Despite progress in defining adult-onset inherited blood-related cancer predisposition syndromes, challenges persist due to the rarity of these syndromes, underperformance in evaluations for FHM, and a lack of clinician awareness or detailed information on familial MPNs and DNA repair deficiency (DNA-RD), particularly *NHEJ1* variants. Overlapping features among these syndromes, phenotypic heterogeneity within the same syndrome, and the absence of consistent diagnostic criteria at presentation further complicate the issue [17].

To address these gaps, we conducted thorough case-based studies on patients suspected of having a genetic predisposition to FHM. Our goal was to understand variant distribution, identify FHM risk factors, explore genetic pathways, and investigate the phenotypic heterogeneity of these rare syndromes. Herein, we present our experience and preliminary findings from the first six years of genetic evaluation and counseling.

## Subjects and methods

### Subjects and cohort

Between February 2018 and February 2024, the hematology unit at Sohag University Hospitals in southern Egypt managed approximately 1,142 patients aged 12 to 89 years. Among these, 780 were confirmed to have hematologic malignancies (HMs). This cohort included 13 out of 357 patients diagnosed with bone marrow failure (BMF) disorders, such as aplastic anemia (AA) and hypoplastic myelodysplastic syndrome (hMDS). Additionally, 8 out of 27 patients were identified with inherited immunodeficiencies, while the remaining 758 patients were diagnosed with various HMs during this period (Fig. S1). To enhance the clarity of patient selection methods, we categorized the initial reasons for suspecting a genetic predisposition to FHM into one of the following criteria. **(1)** Patients with clinicopathologic features of IBMFS or inherited immunodeficiency disorders who developed MDS/HM. **(2)** Patients with HM and a strong family history of malignancy (at least one or more first-degree relatives or two or more second-degree relatives affected by HM). **(3)** Patients with HM and multiple primary malignancies. **(4)** Patients for whom follow-up is recommended due to genetic mutations associated with an elevated risk of HM.

From 66 suspected cases with a potential genetic predisposition to FHM, we identified 33 patients from 19 different Egyptian families for detailed analysis. These cases were used to define the risk factors of FHM and investigate the underlying causes of phenotypic variability.

The diagnosis of IBMFSs was based on several factors, including a family history of hematologic abnormalities, consanguinity, childhood onset of BMF, lack of response to immunosuppressive therapy (IST), and the presence of physical anomalies characteristic of IBMFSs or associated genetic mutations, as previously reported [18]. Following current standards and WHO criteria for classifying myeloid neoplasms [19, 20], hMDS and AA were distinguished from one another.

Comprehensive clinical assessments and investigations were used to describe the phenotypes of all patients**.** Peripheral blood samples were collected various genetic tests, including those for DNA-RD, familial hematopoietic disorders, Shwachman-Diamond syndrome (SDS), telomeropathies, and Fanconi anemia (FA). All patients underwent BM aspirations and biopsies to assess cellularity. Additionally, we reviewed BM pathology and the complete blood count results from their initial hospitalization. Diagnoses of leukemia and lymphoma followed current World Health Organization classification guidelines [21, 22].

For comparative assessments of immunoglobulin levels and lymphocyte subset differences, a control cohort of 25 unrelated healthy Egyptian individuals without clinical symptoms or a family history of BMF or malignancy was included. The study protocol received approval from the Medical Research Ethics Committee of the Faculty of Medicine-Sohag University (Sohag, Egypt). Written informed consent was obtained from all participants or their authorized legal guardians, and all procedures were conducted following the principles outlined in the Declaration of Helsinki.

### Literature search

Since autosomal recessive variants in the *NHEJ1* gene have been reported very rarely, we screened for other patients with *NHEJ1* variants in the literature to provide new clinical and immunological insights on NHEJ1 deficiency based on a newly diagnosed patient with combined immunodeficiency and BMF. We reviewed published cases from 2003 to date using the Human Gene Mutation Database and NCBI—PubMed, utilizing the terms "Cernunnos," "XLF," "XRCC4-like factor," "*NHEJ1*," and "nonhomologous end-joining factor 1". Additional papers were identified by manually searching the reference lists of significant reviews and case series. Table S1 lists the demographic, clinical, immunological, and molecular findings of the reported patients..

Given that the phenotype of NHEJ1 deficiency shares significant similarities with LIG4 deficiency or NBS, we compared 57 patients with NHEJ1 deficiency (including 50 cases from the literature and our 7 new cases) to patients with LIG4 deficient (46 patients) and NBS (216 patients) (Tables S1-S3). This comparison aimed to assess the optimal use of available diagnostic tools and identify parameters that could refine precise diagnosis, predict outcomes, guide treatment options, and facilitate multidisciplinary care for patients with DSBR.

## Methods

To determine the causes of BMF and exclude other causes of FHM, several hematological, biochemical, and molecular investigations were conducted. These included flow cytometry, fluorescence in situ hybridization (FISH), cytogenetic analysis, and a range of laboratory and genetic tests. Testing for FA was performed by diepoxybutane (DEB)-induced chromosomal breakage assay (Fig. S2) was evaluated through gene panel testing and/or whole-exome sequencing (WES) and Sanger analysis. A telomere length (TL) below the age-adjusted first percentile, in the context of a confirmatory clinical history, was considered consistent with a telomere syndrome. Diagnostic approaches were guided by clinical evaluation and laboratory results, depending on suspected causes and available resources.

All cases were screened for possible causes of acquired aplastic anemia (AA), including autoantibodies, viral infections, autoimmune disorders, and serum immunoglobulin levels (IgG, IgA, IgM, and IgE). Mutations in genes known to cause inherited bone marrow failure syndromes (IBMFS) were also investigated. Additional diagnostic tools, such as histological studies of lymph nodes, liver, or spleen, echocardiography, ultrasound and Doppler imaging, magnetic resonance imaging (MRI), and positron emission tomography (PET) scans, were utilized based on suspected underlying causes.

Genomic DNA was extracted from blood cells and buccal mucosa samples using the QIAamp DNA Mini Kit (QIAGEN). RNA was extracted from total leukocytes using the RNeasy Mini Kit (Qiagen) for quantitative and qualitative detection of *BCR-ABL1 major* (p210), *BCR-ABL* p190, and other minor breakpoints. *JAK2* V617F, *JAK2* exon 12, *CALR*, *MPL* mutations, and *BCR-ABL* major and minor breakpoints were detected in peripheral blood white cells, using a real-time polymerase chain reaction assay with hybridization probes/primers designated to detect these mutations, followed by melting curve analysis.

### Flow cytometry

The proportions and lymphocyte count of T cells, B cells, and NK cells, as well as leukemia/lymphoma subtypes, were determined in blood samples and bone marrow (BM) using conjugated mouse anti-human monoclonal antibodies. Data were collected using a FACSCanto II instrument (Becton Dickinson, Franklin Lakes, NJ, USA) and analyzed with the FlowJo software package, version 10.0.7 (Treestar, Ashland, OR, USA).

### Whole exome sequencing and mutation analysis

High molecular weight genomic DNA was extracted from blood and/or buccal mucosa samples. Exome capture was performed using the xGen Exome Research Panel v2 (Integrated DNA Technologies, Coralville, Iowa, USA), and sequencing was conducted using the NovaSeq 6000 (Illumina, San Diego, CA, USA) with 150 bp paired-end reads. Sequencing data were aligned to the GRCh37/hg19 human reference genome using BWA-MEM and processed for variant calling by GATK v.3 [23]. Variants were annotated using the Ensembl Variant Effect Predictor (VEP) and filtered and classified using EVIDENCE [24], following the American College of Medical Genetics and Genomics (ACMG) guidelines [25]. The filtered and classified variant list was manually reviewed by medical geneticists and physicians to identify potential novel IBMF-causing genes, DSBR-related genes, and putative novel genes associated with malignancy susceptibility. The most likely variants explaining the patient’s phenotype were selected for reporting. Most WES analyses were performed at 3billion Laboratory (3billion Inc., Seoul, South Korea), except for family 3, which was analyzed at Kyoto University (Japan) [26].

### Statistical analysis

Statistical analysis was performed using SPSS 22.0 software (SPSS, Inc., Chicago, IL). Figures were generated using the GraphPad Prism software package, version 5.02 (San Diego, CA). Statistical significance was assumed at a *P*-value of < 0.05 in all analyses.

For additional details on diagnostic methods, statistical analysis, and follow-up, refer to the supplementary methods.

## Results

**Fig. 1:**
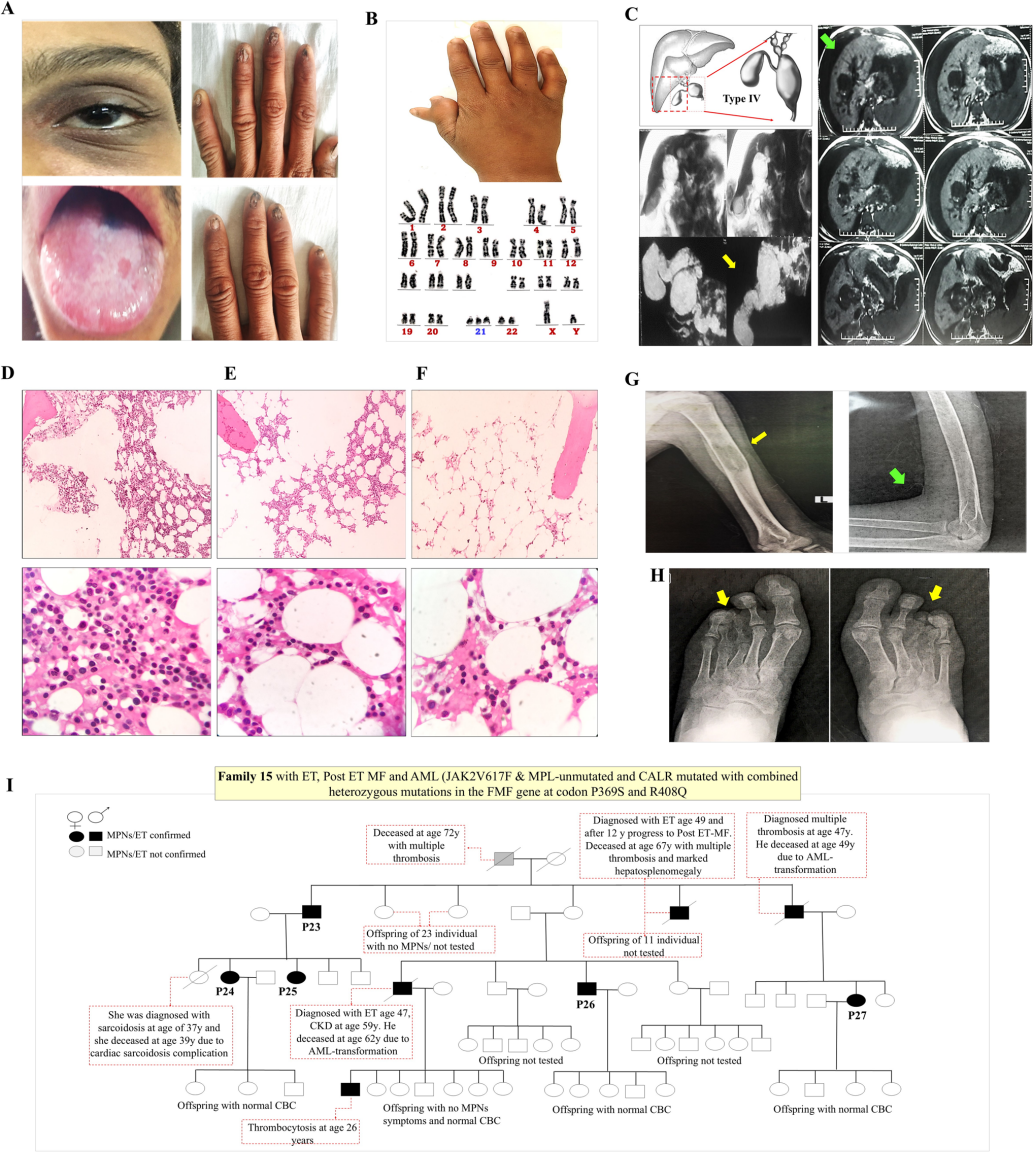
**A** Patient with dyskeratosis congenita (P10) and homozygous *TERT* c.2086 C → T (p.R696C) mutation with compound heterozygous variants in *NOC4L* gene has abnormal brownish skin pigmentation around his eyes (resemble the dark circles around a raccoon’s eyes), and leukoplakia of the tongue with significant nail dystrophy on the hands. **B** Polydactyly in the hand of patient (P14) with Mosaic Down syndrome with 50% normal and 50% trisomy 21 and Fanconi anemia complicated with AML. **C** A 27-year-old female (P12) had DC-like features, choledochal cyst type IV (yellow arrow), alopecia universalis with advanced liver cirrhosis (green arrow), and severe BMF complicated with MDS/AML. **D-F** Representative bone marrow core biopsies result from patients (P14, P15, P16) with Fanconi anemia, revealed hypocellularity (50%, 20%, 10–5%, respectively) without evidence of cellular dysplasia, malignancy, fibrosis, or granulomas. **G** A 19-year-old boy (P21) with recurrent atraumatic fragility humerus fractures with lytic bone lesions (yellow arrows), thin bone, and joint restriction (green arrow), complicated with B-cell ALL. No pathogenic genetic variant could be determined by WES in this patient. **H** A 21-year-old man (P22) had down syndrome features with skeletal abnormalities include scoliosis, third and fourth metatarsal shortening (brachymetatarsia) (yellow arrows), complicated with CML accelerated phase. **I** Pedigree, clinical features, malignancy type and molecular findings of family15 with *CALR* mutation. Abbreviations: CD: cluster of differentiation, CKD; chronic kidney disease, MPNs: myeloproliferative neoplasms, post ET-MF: post essential thrombocythemia myelofibrosis, PNH: paroxysmal nocturnal hemoglobinuria

Out of the initial 357 patients diagnosed with BMF, 205 were excluded from the analysis due to known causes. Among the remaining 152 patients, 53(34.9%) exhibited clinicopathological features consistent with IBMFSs, such as microcephaly, short stature, skeletal or renal abnormalities, and abnormal skin, nails, or hair (Figs. 1A-H, S3, and S4). Within this group, 13(24.5%) patients (11 new, and 2 updated patients [26]) developed HM and had a family history suggestive of FHM. This diverse cohort included patients from 11 families with a median age of 19.5 years (range: 15–42 years) WES and gene panel testing identified mutations in two genes (*TERT*, *DKC1*).

Additionally, among the 27 patients initially diagnosed with inherited immunodeficiency disorders, 11 were suspected of having HM along with features indicative of FHM. Three patients —one with caspase-8 deficiency and two with BTK gene mutations—were subsequently diagnosed with autoimmune lymphoproliferative syndrome (ALPS) or reactive lymphadenopathy with BMF and were excluded from the analysis. The remaining 8 (29.6%) patients, including 6 from the same family with *NHEJ1* mutations and 2 with *LYST* mutations, developed secondary MDS (sMDS)/AML or lymphoma, with a family history suggestive of FHM. This cohort included patients from 2 families, with a median age of 19.0 years (range: 17–24 years).

In a separate group of 758 patients presenting with HM, 49 (6.5%) had a history of hereditary cancer syndromes (HCS), with only 23 (46.9%) of them having a history of FHM. Among these, only 12 patients were included for deeper genetic study, consisting of 10 with myeloproliferative neoplasm (MPNs) and 2 with multiple myeloma (MM) (Fig. S1). This cohort included patients from 6 families, with a median age of 44.5 years (range: 21–73 years). Gene panel testing identified 4 causative mutations (*BCR-ABL p210*, *JAK2 V617F*, *CALR*, and *BRCA2*).

### Phenotypic heterogeneity and links to hematologic disorders among patients with or without DNA repair deficiencies

Interestingly, 16 out of 33 enrolled patients (48.5%) were found to have underlying causes of DNA-RD, including 8(50.0%) patients with FA, 6(37.5%) with DSBD due to homozygous variant in *NHEJ1* gene, and 2 (12.5%) *BRCA2* carriers. In contrast, DNA-RD-negative patients included 10 (58.8%) patients with familial MPNs, 4(23.5%) with DC, 2(11.8%) with Chediak-Higashi syndrome due to homozygous variant in *LYST* gene, and one (5.8%) patient without an identified genetic cause by WES. Patients with DNA-RD were younger (mean age: 24.8 ± 10.0 years, range: 15–44 years) compared to DNA-RD-negative patients (mean age: 34.9 ± 18.6 years, range: 16–73 years). Growth failure, learning disorders, recurrent infections, and endocrinal disorders such as diabetes mellitus (DM) were significantly more prevalent in DNA-RD patients (81.2%, 75.0%, 68.8%, and 56.2%, respectively; *p* < 0.05). The frequency of facial dysmorphism and microcephaly was also significantly higher among DNA-RD patients. Eleven (68.8%) of the 16 DNA-RD patients initially presented with thrombocytopenia, and 14 (87.5%) developed BMF during the disease course. Regular follow-up, including BM aspiration every 18–24 months, revealed that 12 (75.0%) of them progressed to sMDS. In contrast, progression to sMDS was observed in only 4 (23.5%) patients in the DNA-RD-negative subcohort (Table 1). Interestingly, among the 17 patients without DNA-RD, one was diagnosed with AA before progressing to acute lymphoblastic leukemia (ALL). Patient-21, a 19-year-old male, was diagnosed with chronic recurrent multifocal osteomyelitis (CRMO) at age 13. A bone biopsy confirmed CRMO, ruling out malignancy. Two months later, he developed pancytopenia, and a bone marrow biopsy confirmed severe AA. Treatment with naproxen and steroids led to rapid and complete recovery. At age 15, he was diagnosed with Philadelphia chromosome-negative B-cell ALL (Ph[-] B-ALL) and underwent intensive pediatric-style chemotherapy, achieving complete remission. This patient exhibited HCS/FHM without identifiable genetic causes predisposing to FHM. His first cousin had follicular lymphoma at age 27, his nephew had Ph[-] B-ALL at age 11, his niece passed away at age 4 due to multiple congenital anomalies and a central nervous system (CNS) tumor, and his cousin’s son died at age 14 from severe BMF.

**Table 1:** Comparison of the characteristics of patients with significant family history of hematologic malignancies, who subsequently develop hematologic malignancies with and without DNA repair disorders

		Patients with hematologic malignancies (with significant family history of hematologic malignancies)		*P* value
		With DNA RD -Group*n* = 16	Without DNA RD-Group *n* = 17	
Sex	Male	8(50.0%)	10(58.8%)	0.389
	Female	8(50.0%)	7(41.2%)	
Age, Mean ± SD (Years)		24.8 ± 10.0	34.8 ± 18.4	0.060
Age, at first diagnosis		20.4 ± 9.6	20.0 ± 17.5	0.151
History of consanguinity		16(100%)	16(94.1%)	0.527
Significant medical History				
Growth failure		13(81.2%)	1(5.8%)	**< 0.001**
Recurrent infections		11(68.8%)	2(11.8%)	**0.001**
Learning disorders		12(75.0%)	3(17.6%)	**0.001**
Endocrinal disorders*		9(56.2%)	2(11.8%)	**0.007**
Low serum immunoglobulins		6(37.5%)	2(11.8%)	0.081
Significant clinical examinations				
Facial dysmorphism		8(50.0%)	1(5.8%)	**0.005**
Microcephaly		8(50.0%)	0(0.0%)	**0.001**
Other congenital anomalies		7(43.8%)	6(35.3%)	0.872
Family history of abnormalities		13(81.2%)	9(52.9%)#	0.028
Family history of malignancy				
Solid malignancy		15(93.8%)	9(52.9%)	0.008
Myeloid malignancy		9(56.2%)	10(58.8%)	0.620
Lymphoid malignancy		6(37.5%)	2(11.8%)	0.110
Breast cancer		6(37.5%)	4 (23.5%)	0.457
Lung cancer		6(37.5%)	0(0.0%)	**0.006**
Prostate cancer		3(18.8%)	1(5.8%)	0.323
Squamous cell carcinoma		2(12.5%)	0(0.0%)	0.241
GIT or liver malignancy		2(6.2%)	3(17.6%)	0.604
MPNs		0(0.0%)	9(52.9%)	**0.001**
Final diagnosis		6(37.5%) DSBD, 8(50.0%) FA, 2(12.5%) *BRCA2* mutation carriers	10(58.8%) MPNs, 4(23.5%)DC 2 (11.8%)CHS, 1 (5.8%)AA	-
Disease progression and complications				
Thrombocytopenia first presentation		11(68.8%)	4 (23.5%)	**0.004**
Progression to BMF		14(87.5%)	7(41.2%)	**0.005**
Progression to secondary MDS		12(75.0%)	5(29.4%)	**0.020**
Age, at hematological malignancy		24.2 ± 9.8	30.0 ± 17.6	0.338
Type of final hematological malignancy		7(43.8%) AML, 5(31.2%)MDS 2(12.5%)MM, 2(12.5%)DLBCL	4(23.5%)MDS, 5(29.4%)ET, 2(11.8%)MF, 2(11.8%)AML 5(29.4%) (ALL, Post ET-MF, CML, PV, lymphoma each one case)	-
Myeloid malignancy		12(75.0%)	15(88.2%)	0.395
Lymphoid malignancy		2(12.5%)	2 (11.8%)	0.648
Acute leukemia/MDS-EB		9(56.2%)	3(17.6%)	**0.031**
Prognosis (Deaths)		7(43.8%)	4(23.5%)	0.274

*Diabetes mellitus or hypothyroidism, ¶Group with DNA RD …9 with endocrinal disorders, 2 with family history of BMF, 2 with family history with multiple congenital anomalies

^#^Group without DNA RD …2 with endocrinal disorders, one with family history of BMF, 5 with family history of multiple congenital anomalies and one with multimember of his family with liver cirrhosis

Abbreviation: *AA* Aplastic anemia, *ALL* Acute lymphoblastic leukemia, *AML* Acute myeloid leukemia, *CHS* Chediak-Higashi syndrome, *CML* Chronic myeloid leukemia, *DC* Dyskeratosis congenital, *DLBCL* Diffuse large B-cell lymphoma, *DM* Diabetes mellitus, *DNA RD* DNA repair disorders, *DSBR* Double-strand break repair disorders, *ET* Essential thrombocythemia, *FA* Fanconi anemia, *MDS* Myelodysplastic syndromes, *MDS-EB* Myelodysplastic syndromes with excess blasts, *MDS-MLD* MDS with multilineage dysplasia, *MM* Multiple myeloma, *MPNs* Myeloproliferative neoplasms, *Ph(* +*)/* Philadelphia chromosome (positive), *SCC* Squamous cell carcinoma

### Association between DNA repair deficiency and MDS/Acute leukemia evolution

The occurrence of malignancy was earlier in patients with DNA-RD (24.2 ± 9.8 years, age range 13–43 years) compared to patients without DNA-RD (30.0 ± 16.5 years, age range 15–66 years), with a higher tendency toward myeloid malignancies. Clonal evolution and the frequent development of AML or MDS with excess blasts (56.2%) were common complications among DNA-RD patients during their teens or young adulthood. Additionally, lung and breast cancer (37.5%) were the most prevalent solid malignancies among family members of DNA-RD patients. Squamous cell carcinoma (SCC) was exclusively observed in patients with Fanconi anemia (FA) and their family members. In contrast, MPNs (58.8%) more prevalent in patients without DNA-RD (Table 1). The detailed clinical features of patients with or without DNA-RD are provided in (Tables 2 and 3).

**Table 2:** Clinical and laboratory characteristics of patients with DNA repair disorders, inherited immunodeficiency disorders, BMFSs, or a significant family history of hematologic malignancies, who subsequently develop hematologic malignancies

Classification	Patient ID / Family	Age/Sex	Significant medical history & clinical examinations	Diagnosis and age at diagnosis	Hematological malignancy	Family history of malignancy or abnormalities	Cytogenetics & testing performed
Double-strand break repair disorders	P1 Family-1 (III-B1)	19/M	Multiple consanguineous marriages Growth failure, Microcephaly, Facial dysmorphism (long nose, large ears), Learning disorders, Severe combined immunodeficiency, Recurrent infections (Bacterial, viral, and fungal) Sensitivity to ionizing radiation, All the 6 patients developed thrombocytopenia for a range (5 to 15 years) prior to pancytopenia and BMF diagnosis P3&P4 had a higher frequency of recurrent viral infections than other patients P4 had EBV acute infection prior to LPD diagnosis	SCID/DM at the age of 11y, Sever AA phase age 15y	AML diagnosis age 19y (deceased)	Paternal grandfather died of lung cancer (unknown details), Endocrinal manifestations (type I or type II DM and hypothyroidism) were diagnosed between carriers Proband- (III-B5) who was without symptoms until age 10y, was diagnosed with SCID during family screening At age 11, the manifestations of recurrent infection started, and he was diagnosed with ADHA without BMF SCID was observed in 7 of the family members 6 only developed BMF and hematological malignancies	Normal karyotyping for all patients , By WES: Homozygous canonical splice site variant, c. 530-2A > T, at the intron 4 of *NHEJ1* gene (NM_ 024782.3) Protein:NP_079058.1:p DEB assay negative
	P2 Family-1 (III-B2)	22/F		ITP age 5y, steroid induced DM 5y, SCID age 18y, Type I DM after 16y free from DM, Sever AA phase age 18y	sMDS-MLD age 22y following AA phase diagnosis		
	P3 Family-1 (II-6)	24/M		SCID/DM at the age of 14y, Sever AA phase age 18 y	DLBCL age 23y (deceased)		
	P4 Family-1 (III-A1)	19/M		SCID at the age of 15y, AA/ hMDS, DM age 16y	DLBCL age 19y (deceased)		
	P5 Family-1 (III-C1)	18/F		SCID at the age of 12y, Non-sever AA phase age 14y	sMDS with excess blasts RAEB-2 age 18y (deceased)		
	Family-1 (II-7) P6	21/F		SCID at the age of 14y, DM, BMF age 17 years	AML age 21y (deceased)		
Inherited immunodeficiency disorders	P7 Family-2	17/M	Consanguinity present, P7 and P8 are identical twins who have oculocutaneous albinism (photosensitivity& silvery hair), Recurrent sinopulmonary infections were frequent in P7 while his brother P8 has recurrent skin infections (deep subcutaneous abscesses) P8 has neurological manifestations (Muscle weakness, Intellectual disability, cerebellar ataxia, nystagmus)	Chediak-Higashi syndrome age 14y, At age 15y he developed persistent anemia, LPD age 16y (All lab,, BMA, BMB and LN biopsies excluded HLH/ accelerated phase)	During HSCT preparation, he deceased at age 17y due to lymphoma associated hemophagocytic syndrome	Paternal grandfather died at age 62y with cancer of unknown primary, Maternal aunt—breast cancer, Sister with thrombocytopenia and her son deceased age 2y with multiple congenital anomalies	46, XY, By WES: Homozygous pathogenic variant was identified in *LYST gene*, c 6712C > T, (NM_ _0000814) Protein: NP_000072.2: p.Arg2238Ter DEB assay negative
	P8 Family-2	19/M		Chediak-Higashi syndrome age 14y, At age 18y, he developed refractory thrombocytopenia	MDS-SLD at age 19y		
Bone marrow failure disorders	P9 Family-3	25/F	Consanguinity present, Nail dystrophy, osteopenia, bilateral 1st hole type ribs, high serum IgE level, B and NK cytopenia, with inverted CD4/CD8 ratio were detected in P9 and P10, P10 only has raccoon eyes, right third fork type rib with extensive lytic osseous lesions including the humerus, pelvic, femurs, and tibial bones, P9 has lumber scoliosis	Dyskeratosis congenita, very severe AA phase age 21y, Type II DM diagnosis age 24y	DC-sMDS-MLD diagnosis age 25y evolving from very severe BMF	One brother sustained an atraumatic right tibial fracture at the age of 18 y, followed 6 months later by a second atraumatic left tibial fracture. This brother developed pancytopenia and severe BMF at age 20y, Father and mother with DM, osteopenia, and premature hair greying, at age 28y, & 30y, Grandmother with breast cancer	46, XX, DEB assay negative, < 1% telomere length, By WES: Germline homozygous *TERT* c.2086 C → T (p.R696C) mutation with compound heterozygous variants in *NOC4L* gene
	P10 Family-3	16/M		Atraumatic fragility right femoral fracture at the age of 12y, followed 2 months later by a second atraumatic left femoral fracture Thrombocytopenia at age 12y then pancytopenia (non-severe AA phase)/DC age 12.5y	DC-sMDS-MLD diagnosis age 16y evolving from BMF		
	P11 Family-4	20/M	Consanguinity present, alopecia, skin pigmentation, Nail dystrophy, osteopenia	Thrombocytopenia since age 11y, Pancytopenia, severe AA phase age 14y, Liver cirrhosis age 14y	DC-sMDS-MLD diagnosis age 19y	Brother with hMDS age 16y, 3 Uncles with HCC age 49y, 56y,and 62y, Grandfather with HCC age 59y	46, XY DEB assay negative *DKC1*/ exon 3: c.145A > T missense substitution p.Thr49Ser
	P12 Family-5	27/F	Consanguinity present, alopecia universalis, nail dystrophy, skin pigmentation, oral leukoplakia,, choledochal cyst type IV	Pancytopenia since age 21y, advanced liver cirrhosis diagnosis age 24y	MDS/AML diagnosis age 27y	Father with BMF deceased age 46y, Paternal grandfather with GIT malignancy	46, XX DEB assay negative Clinical conundrum was strongly suggested DC
	P13 Family-6	28/M	Consanguinity present, short stature, skin pigmentation, microphthalmia, small optic discs, erectile dysfunction with hypospermia, FA diagnosis after SSC diagnosis when patient was referred to hematology due to pancytopenia during routine investigations	Fungating mass left ring finger amputated (SCC) with LN metastasis and BMF age 26y He was treated by Paclitaxel/carboplatin age complicated with BMF toxicity, then Paclitaxel only low dose	FA-hMDS-SLD age 26y (before chemotherapy) FA-hMDS-MLD diagnosis age 28y (after 11 months of chemotherapy)	Sister with FA-BMF age 26y & SCC of the tongue age 32y, Grandfather with prostate Cancer	46, XY DEB assay positive Clinical conundrum was strongly suggested FA
	P14 Family-7	16/M	Consanguinity present, Short stature, polydactyly, hypo and hyperpigmented skin areas, learning difficulty	Mosaic Down syndrome, BMF diagnosis/Fanconi anemia age 11y	FA-AML diagnosis age 16y	Brother with AML age 13(deceased), Mother with lifetime thrombocytopenia	47,XY, + 21[10] 46, XY[10] DEB assay positive
	P15 Family-8	15/F	Consanguinity present, short stature, recurrent infection, café au lait patches, learning difficulty	Anemia since birth Fanconi anemia/BMF diagnosis age 7y	FA-sMDS-MLD diagnosis age 13y	Paternal grandmother with gastric cancer, Great grandfather with prostate Cancer	Cytogenetic (NA) DEB assay positive
	P16 Family-9	18/F	Consanguinity present, P16 and P17 are fraternal twins who have microcephaly. short stature, recurrent infection, Café au lait patches, learning difficulty P16 has bilateral parenchymal kidney disease with elevated renal function while P17 has unilateral renal hypoplasia	Anemia age 7y FA-BMF diagnosis age 13y CKD age 16y Hemodialysis age 17y	FA-MDS/AML diagnosis age 17y (deceased due to TRM)	Maternal grandmother with genital cancer, Mother died with cancer of unknown primary,	DEB assay positive P16 cytogenetic results during FA-MDS/AML phase showed 7q deletion (7q −) (FISH) P17 cytogenetic results 46, XX
	P17 Family-9	18/F		Anemia age 8y FA-BMF diagnosis age 17y CKD age 18y	FA-sMDS-MLD diagnosis age 18y		
	P18 Family-10	17/M	Consanguinity present, Low set ears, skin hypopigmentation, hepatomegaly, unilateral pelvic kidney	Thrombocytopenia age 15y Fanconi anemia/BMF diagnosis age 16y	FA-sMDS with excess blasts age 17y, after 3 months FA-AML transformation	First cousin with AML age 14y Second cousin with oral squamous cell carcinoma age 21y	45,XY,−7[9]/46,XY[6] (at time of AML) DEB assay positive
	P19 Family-11	42/M	Consanguinity present, Overweight, skin hypopigmentation, mild hepatomegaly	Thrombocytopenia age 25y Fanconi anemia/BMF diagnosis age 27y	FA-sMDS-MLD with excess blasts age 41y AML age 42y	Daughter died of BMF age 2y, her clinical features was strongly suggested VACTERL-H (vertebral defects, ASD,, unilateral renal agenesis, lower limbs deformities + hydrocephalus) Brother died of AML age 43y, Sister with breast cancer at age 32y	46, XY[20] (at time of BMF age 28y) 47,XY, + 21[6] 46, XY[14] (at time of sMDS-MLD) DEB assay positive
	P20 Family-12	35/F	Consanguinity present, Ventricular septal defect Recurrent skin infections and abscesses, Recurrent respiratory tract infection	Persistent leukopenia and anemia Thrombocytopenia age 23y Fanconi anemia/BMF diagnosis age 26y	FA-sMDS-MLD age 30y FA-AML transformation age 34.5 years Treatment by azacitidine venetoclax protocol	Maternal grandmother died of breast cancer Brother died of AML at age 24y	46, XX[20] (at time of MDS age 30y) DEB assay positive t(16;16),inv(16) negative t(8;21) (q21;q22) AML1/ETO negative
	P21 Family-13	19/M	Multiple consanguineous marriages Painful inflammatory bilateral symmetrical bony lesions include the metaphysis of long bones age 13y, bone biopsy excluding malignancy and confirmed chronic nonbacterial osteomyelitis with negative autoimmune profiles	CRMO was diagnosed at the age of 13y, followed 2 months later by pancytopenia and BMB confirmed severe AA Treatment by naproxen and steroid showed quickly complete recovery	Ph(-) B-cell ALL age 15y Treatment by pediatric-style intensive chemotherapy regimen showed complete remission	First cousin with follicular lymphoma age 27y Nephew with Ph(-) B-ALL age 11y Niece with multiple congenital anomalies and CNS tumor Cousin’s son with AA age 14y	46, XY Ph (negative) Bcr–Abl p210 (negative) Bcr–Abl p190 (negative) By WES: No clinically significant variant was identified DEB assay negative
Myeloproliferative neoplasms	P22 Family-14	21/M	Consanguinity present, Down syndrome features (Brachycephaly, slanted eyes, flat nose, folded ears, abnormal teeth, short neck, short, broad hands and brachymetatarsia with atrioventricular septal defect) Learning disorders, Marked splenomegaly 22 cm	Down syndrome diagnosis age 2y, Type 1 DM age 14y Persistent anemia age 18y CML (WBCs 256.9 × 10^9^/L, Hb 7.6 g/dL, platelets 136 × 10^9^/L) age 19y	CML chronic phase age 19y, treatment by Imatinib CML accelerated phase age 21y, treatment by Nilotinib Deceased at age 21y before HSCT	His brother (down syndrome) developed leukocytosis and was diagnosed with CML Ph( +) at age 15y, After 11 months, CML progress to AML, and he deceased at age 16y due to AML-TRM Maternal grandmother died of AML	47,XY, + 21 BCR-ABL p210 (positive) BCR-ABL Quantitative (89.43%)
	P23 Family-15	73/M	Consanguinity present, P24 and P25 are daughters of P23 P26 and P27 are first cousins of P24, and P25 P26 and P27 are first cousins also P23 has moderate splenomegaly. and heterozygous mutation in the FMF gene at codon P369S without FMF attacks P24 has DVT and heterozygous mutation in the FMF gene at codon P369S without FMF attacks P25 has combined heterozygous mutations in the FMF gene at codon P369S and R408Q, without FMF attacks, but she has high ESR 1st hour 93, CRP: normal, Serum amyloid A:normal, and normal immune profile P26 and P27 have moderate splenomegaly and DVT	ET, acrofacial vitiligo, IHD at age 62y Post-ET myelofibrosis age 72y	ET treatment by aspirin + hydroxyurea Post-ET MF age 72y, treatment by Ruxolitinib	Brother of P23 was diagnosed with ET age 49 and after 12 y progress to Post ET-MF. Deceased at age 67y with multiple thrombosis and marked hepatosplenomegaly Oldest daughter of P23 was diagnosed with sarcoidosis at age of 37y and she deceased at age 39y due to cardiac sarcoidosis complication Nephew of P23 was diagnosed with ET age 47 and CKD at age 59y after 12y and he deceased at age 62y due to AML-transformation Cousin’s son of P23 was diagnosed with ET age 22 Father of P27 (brother of P23) was diagnosed with multiple thrombosis at age 47y, and he deceased at age 49y due to AML-transformation	46, XY *CALR* mutation exon 9 ( +) *JAK2* V617F mutation (-) *MPL* mutation exon 10 (-) BCR-ABL Quantitative (0)
	P24 Family-15	34/F		Essential thrombocythemia (WBCs 26.3 × 10^9^/L, Hb 12.9 g/dL, platelets 1175 × 10^9^/L) age 29y	Essential thrombocythemia on hydroxyurea + anticoagulant		46, XX *CALR* mutation exon 9 ( +) *JAK2* V617F mutation (-) *MPL* mutation exon 10 (-) BCR-ABL Quantitative (0)
	P25 Family-15	27/F		Essential thrombocythemia (WBCs 17.8 × 10^9^/L, Hb 11.9 g/dL, platelets 815 × 10^9^/L) age 26y	Essential thrombocythemia on aspirin		46, XX *CALR* mutation exon 9 ( +) *JAK2* V617F mutation (-) *MPL* mutation exon 10 (-) BCR-ABL Quantitative (0)
	P26 Family-15	51/M		ET, DVT age 36y Post-ET myelofibrosis age 48y AML, age 51y	ET then Post-ET MF treatment by anticoagulant + hydroxyurea Deceased at age 51y with AML		46, XY *CALR* mutation exon 9 ( +) *JAK2* V617F mutation (-) MPL mutation exon 10 (-) BCR-ABL Quantitative (0)
	P27 Family-15	42/F		Essential thrombocythemia (WBCs 19.2 × 10^9^/L, Hb 13.1 g/dL, platelets 1482 × 10^9^/L) age 35y	Essential thrombocythemia on hydroxyurea + anticoagulant +		46, XX CALR mutation exon 9 ( +) JAK2 V617F mutation (-) MPL mutation exon 10 (-) BCR-ABL Quantitative (0)
	P28 Family-16	56/F	Consanguinity present, Moderate splenomegaly	IHD & Essential thrombocythemia (WBCs 16.2 × 10^9^/L, Hb 15.1 g/dL, platelets 1324 × 10^9^/L), with normal erythropoietin level age 51y Renal cell carcinoma (Radical nephrectomy& pembrolizumab) age 55y	Essential thrombocythemia on aspirin + hydroxyurea	Mother was diagnosed with ET (*JAK2* mutation positive), IHD at age 68 y and treated by aspirin + hydroxyurea Maternal grandmother died with multiple thrombosis	46, XX JAK2 V617F mutation ( +) CALR mutation exon 9 (-) MPL mutation exon 10 (-) BCR-ABL Quantitative (0)
	P29 Family-17	51/M	Multiple consanguineous marriages Moderate splenomegaly Left side hemiparesis due to recurrent ischemic stroke	HBT (Hb A2 5.6%, Hb F 1.4%) age 24y Polycythemia (WBCs 16.7 × 10^9^/L, Hb 19.2 g/dL, platelets 678 × 10^9^/L) age 49y	Polycythemia vera on hydroxyurea and venesection	His son (age 14y) was diagnosed with β-thalassemia major, and splenectomy was done at age 8y. His wife (HBT) deceased at age 34y with gastric cancer His brother with *JAK2(* +)PV Multiple family members have β-thalassemia major/intermedia or trait Sister of maternal grandmother deceased with lymphoma	46, XY JAK2 V617F mutation ( +) CALR mutation exon 9 (-) MPL mutation exon 10 (-) BCR-ABL Quantitative (0)
	P30 Family-18	45/F	Multiple consanguineous marriages P 30 and P31 are daughter and mother	Budd chiari syndrome with normal CBC & JAK2 mutation (positive) Occult MPNs age 36y Overt MPNs (WBCs 41.1 × 10^9^/L, Hb 18.3 g/dL, platelets 644 × 10^9^/L) age 44y	Primary myelofibrosis, stage IV fibrosis on Hydroxyurea then was treated with Ruxolitinib	Maternal grandmother deceased with PV and multiple thrombosis Maternal grandmother’s cousin deceased with leukemia	46, XX JAK2 V617F mutation ( +) BCR-ABL Quantitative (0) CALR mutation exon 9 (-) MPL mutation exon 10 (-) Prothrombotic workup (-) APS- antibodies (-) PNH (-)
	P31 Family-18	70/F		Primary myelofibrosis (WBCs 15.1 × 10^9^/L, Hb 15.3 g/dL, platelets 882 × 10^9^/L) age 66y	Primary myelofibrosis stage III fibrosis on Ruxolitinib		46, XX JAK2 V617F mutation ( +) BCR-ABL Quantitative (0)
Multiple myeloma	P32 Family-19	44/M	Multiple consanguineous marriages P32 and P33 are brother and sister	Partial compression fractures of upper-end plates of several vertebraes	MM age 43y with 62% BM plasma cells, IgG-lambda paraproteinaemia of 7.11 g/l	Multiple family members with breast cancer Mother died with breast cancer	P32 cytogenetic 46, XY P33 cytogenetic 46, XX *BRCA2* mutation carriers
	P33 Family-19	41/F		Pathological fracture due to lytic lesion of the proximal femur	MM age 39y with 45% BM plasma cells, IgG-lambda paraproteinaemia of 6.12 gm/dl		

*AA* Aplastic anemia, *ADHA* Attention deficit hyperactivity disorder, *ALL* Acute lymphoblastic leukemia, *AML* Acute myeloid leukemia, *APS* Antiphospholipid syndrome, *ASD* Atrial septal defect, *DC* Dyskeratosis congenital, *DLBCL* Diffuse large B-cell lymphoma, *BMA* Bone marrow aspiration, *BMB* Bone marrow biopsy, *BMFS* Bone marrow failure syndromes, *BOS* Bronchiolitis obliterans syndrome, *CML* Chronic myeloid leukemia, *CNS* Central nervous system, *cGvHD* Chronic graft versus host disease, *CKD* Chronic kidney disease, *CRMO* Chronic recurrent multifocal osteomyelitis, *CyA* Cyclosporine A, *DC* Dyskeratosis congenital, *DEB* Diepoxybutane, *DM* Diabetes mellitus, *DSBR* Double-strand break repair disorders, *EBV* Epstein-Barr virus, *ET* Essential thrombocythemia, *FA* Fanconi anemia, *FISH* Fluorescence in situ hybridization, *FMF* Familial Mediterranean fever, *Hb* Hemoglobin, *HBT* Heterozygous beta-thalassemia, *HCC* Hepatocellular carcinoma, *HLH* Hemophagocytic lymphohistiocytosis, *hMDS* Hypoplastic MDS, *HSCT* Hematopoietic stem cell consultation, *IHD* Ischemic heart disease, *LN* Lymph node, *LPD* Lymphoproliferative disorder, *MDS* Myelodysplastic syndromes, *MDS-MLD* MDS with multilineage dysplasia, *MDS-SLD* MDS with single lineage dysplasia, *MM* Multiple myeloma, *MPNs* Myeloproliferative neoplasms, *NA* Not available, *NK* Natural killer, *PBSCT* Peripheral blood stem cell transplant, *Ph(* +*)/(-)* Philadelphia chromosome (positive/negative), *PNH* Paroxysmal nocturnal hemoglobinuria, Post Essential thrombocythemia myelofibrosis, *PRES* Posterior reversable encephalopathy syndrome, *sMDS* Secondary myelodysplastic syndromes, *SCC* Squamous cell carcinoma, *SCID* Severe combined immunodeficiency, *TMR* Treatment-related mortality, *TPO-RA* Thrombopoietin, *VACTERL* Vertebral defects, anorectal malformations, cardiovascular defects, tracheoesophageal defects, renal anomalies, and limb deformities, *WES* Whole exome sequencing, *y* years

**Table 3:** Clinical features, laboratory and immunologic work up of family with the *NHEJ1* mutation

Patient’s ID	Case III-B1	Case III-B2	Fraternal twins		III-B3	III-B4	III-B7	II-4	II-5
			Case III-B5	III-B6					
Age (years) / Sex	19 / Male	22 / Female	12 / Male	12 / Male	19 / Male	18 / Male	**3** / Male	42 / Female	50 / Male
Family position	1st son	1st daughter	4th son	5th son	2nd son	3rd son	6th son	Mother	Father
Growth failure	Yes	Yes	Yes	No	No	No	No	No	No
Neurologic disorders	No	No	ADHA	No	No	No	No	No	No
Learning disorder	Yes	Yes	Yes	No	No	No	No	No	No
Type of repeated infection	RTI/ Diarrhea	Diarrhea/UTI	Diarrhea	No	No	No	No	No	No
Diabetes mellitus	Type-I	Type-I	No	No	Type-I	No	No	Type-II	Type-II
Physical features									
Microcephaly	Yes	Yes	Yes	No	No	No	No	No	No
Long nose	Yes	Yes	Yes	No	Yes	No	No	Yes	Yes
Large ears	Yes	Yes	Yes	No	No	No	No	No	No
Height (cm)	143	145	139	150	175	173	95	172	178
Weight (Kg)	42	41	32	46	89	83	19	106	103
BMI *	20.5	19.5	16.6	20.4	29.1	27.8	21.1	35.8	32.5
Laboratory findings									
WBCs (cells/mm^3^)	7300	1200 ↓↓	5800	8800	6200	8020	5700	9600	10,900
Neutrophil, % cells/mm^3^	60%, 4380	42%, 504↓	69.5%, 4031	53.4%, 4699	61.5%, 3813	56.6%, 4539	68%, 3876	64.1%, 6153	62.5%, 6813
Lymphocyte, %, cells/mm^3^	31%, 2263	51%, 612↓	17%, 1003↓	34.6%, 3004	30.1%, 1866	31.9%, 2558	23.1%, 1317	27.2%, 2611	30.3%, 3302
Monocyte %, cells/mm^3^	8.0%, 584	4.9%, 58.8	11.2%, 650	7.4%, 651	6.2%, 384	4.9%, 393	6.8%, 388	3.9%, 375	5.9%, 643
Eosinophils %, cells/mm^3^	1.0%, 73	0.2%, 2.4	1.7%, 99	3.2%, 282	1.3%,80.6	3.1%, 249	0.8%, 45.6	4.2%, 403↑	0.9%, 98
Basophils %, cells/mm^3^	0%, 0.0	1.1%,, 13.2	0.3%, 17.4	1.45%, 128	0.8%, 49.6	0.5%, 40.1	1.24%, 70.7	0.6%, 57.6	0.3%, 32.7
RBCs(3.8–4.8 × 10^6^/ uL)	2.7 ↓	1.8 ↓	4.41	4.9	5.4	5.6	4.48	4.6	5.4
Hemoglobin (12–15 g/dL)	9.8 ↓	4.5 ↓	13.0	11.8	14.3	13.4	12.3	11.2	14.9
HCT(36.0–46.0)%	29.7↓	15.9↓	38.9	38.4	44.3	43.6	38.0	36.3	45.8
MCV(77–97) fL	108 ↑	80.9	88.2	77.8	82.7	77	84	78	84.7
MCH(27–32)pg	35	24.7↓	29.5	23.9	26.6	23.4	27.5	24.1	27.5
MCHC (31.5–34.5) g/dL	32	30.9↓	33.4	30.7	32.3	30.7	32.4	30.8	32.5
RDW	15.9	20 ↑	12.6	12.6	11.2	12.4	11.9	13.4	13.3
Platelets (× 10^3^ cells/mm^3^)	41↓↓	2↓↓↓	231	288	316	205	185	311	281
MPV	8.4	10.2	9.9	8.2	7.7	9.19	6.5	7.4	11.9
Reticulocytes (0.5%−2.5%)	0.4↓	0.2 ↓↓	1.7 ↔	2.1 ↔	1.3 ↔	1.6 ↔	0.9 ↔	1.2 ↔	1.5 ↔
IgG (mg/dL)	208↓↓	1241 ↔	618 ↓	900.8 ↔	915.1 ↔	1165.7 ↔	1026.1 ↔	1063 ↔	1049 ↔
IgM (mg/dL)	18 ↓↓	168.9 ↔	47.9 ↓	72.3 ↔	63.1 ↔	64.4 ↔	76.7 ↔	92.48 ↔	83.9 ↔
IgA (mg/dL)	16 ↓	45.3 ↓	20.4 ↓	135.8 ↔	128.7 ↔	147.0 ↔	87.5 ↔	276.5 ↔	181.3 ↔
IgE (IU/mL)	10 ↔	3.7 ↔	7.8 ↔	1664↑↑↑	130.5↑	1737↑↑↑	1525↑↑↑	696.6↑↑	34.95 ↔

Age, height, weight, and BMI ––at last follow up (writing of the manuscript)

*Standing height using a portable stadiometer was measured to the nearest millimeter and weight was measured using an electronic scale to the nearest 100 g. Body mass index (BMI) was computed by dividing weight in kilograms by height in meter squares. Subsequently, the height, weight and BMI were converted to Z scores using Egyptian/WHO normative data

*ADHD* Attention-deficit/hyperactivity disorder, *UTI* urinary tract infection, *RTI* respiratory tract infections, *BMI* Body mass index, *p* percentile, *WBCs* white blood cells, *RBCs* red blood cells, *Hb* hemoglobin, *HCT* hematocrit, *MCV* mean corpuscular volume, *MCH* mean corpuscular hemoglobin, *MCHC* mean corpuscular hemoglobin concentration, *Ig* immunoglobulin

### Molecular and clinical profiles in familial myeloproliferative neoplasms

Over a six-year period at our institution, we identified 23 patients (7.2%) from six families among 321 patients diagnosed with MPNs. All these 6 families had at least two relatives affected by MPNs. Of these patients, 19 (82.6%) had a first-degree relative with MPNs, while 4 (17.4%) had a second-degree relative affected. The most frequent diagnosis among participants was essential thrombocythemia (ET, *n* = 12, 52.2%), followed by myelofibrosis (MF, *n* = 7, 30.4%), polycythemia vera (PV, *n* = 2, 8.7%), and chronic myeloid leukemia (CML, *n* = 2, 8.7%). Driver mutations identified included *CALR* exon 9 variants in 13 patients (56.5%), *JAK2* V617F in 9 patients (39.1%), and *MPL* W515L/K in none (0%). All molecular defects were confirmed to be somatic through analysis of DNA from non-hematopoietic tissues. Two patients (8.7%) were triple-negative for these mutations. The two CML cases exhibited *BCR-ABL* rearrangement and an additional copy of chromosome 21.

A complete blood cell count was performed on apparently healthy relatives to identify early asymptomatic MPN phenotypes. The MPN phenotype within each family was homogeneous. While the overall incidence of malignant disorders did not differ significantly between familial and sporadic MPN cases, malignancies were more frequent in patients with familial disease. Three families demonstrated associations between MPNs and other hematological malignancies (e.g., AML, lymphoma) or solid tumors (e.g., gastric and renal cell carcinoma).

Molecular investigations of 10 patients from five families included in this study failed to identify germline mutations in *TET2, MPL,* or *THPO*, that could be associated with hereditary erythrocytosis, thrombocytosis, neutrophilia or familial MPNs.

In one family (Family 15), eight of the ten affected members had ET, and two had post-ET myelofibrosis. All were positive for *CALR* Exon 9 mutations and negative for *JAK2 V617F,* and *MPL*. The index case (P23) developed post-ET myelofibrosis a decade after their ET diagnosis. Two daughters were diagnosed with ET in their late 20 s. Additionally, the index case’s brother and two nephews were diagnosed with AML in their 40 s and 50 s (Fig. 1I). Screening for other germline mutations identified heterozygous FMF gene mutations (P369S) in P24 and combined heterozygous P369S and R408Q FMF mutations in P25, although no FMF symptoms were observed.

A limitation of this study is the lack of population-level analysis of germline *JAK2* gene risk variants and other MPN-predisposition alleles.

#### Phenotypic heterogeneity of *NHEJ1* variants in one family and links to MDS/ leukemia and lymphoma susceptibility

**Fig. 2:**
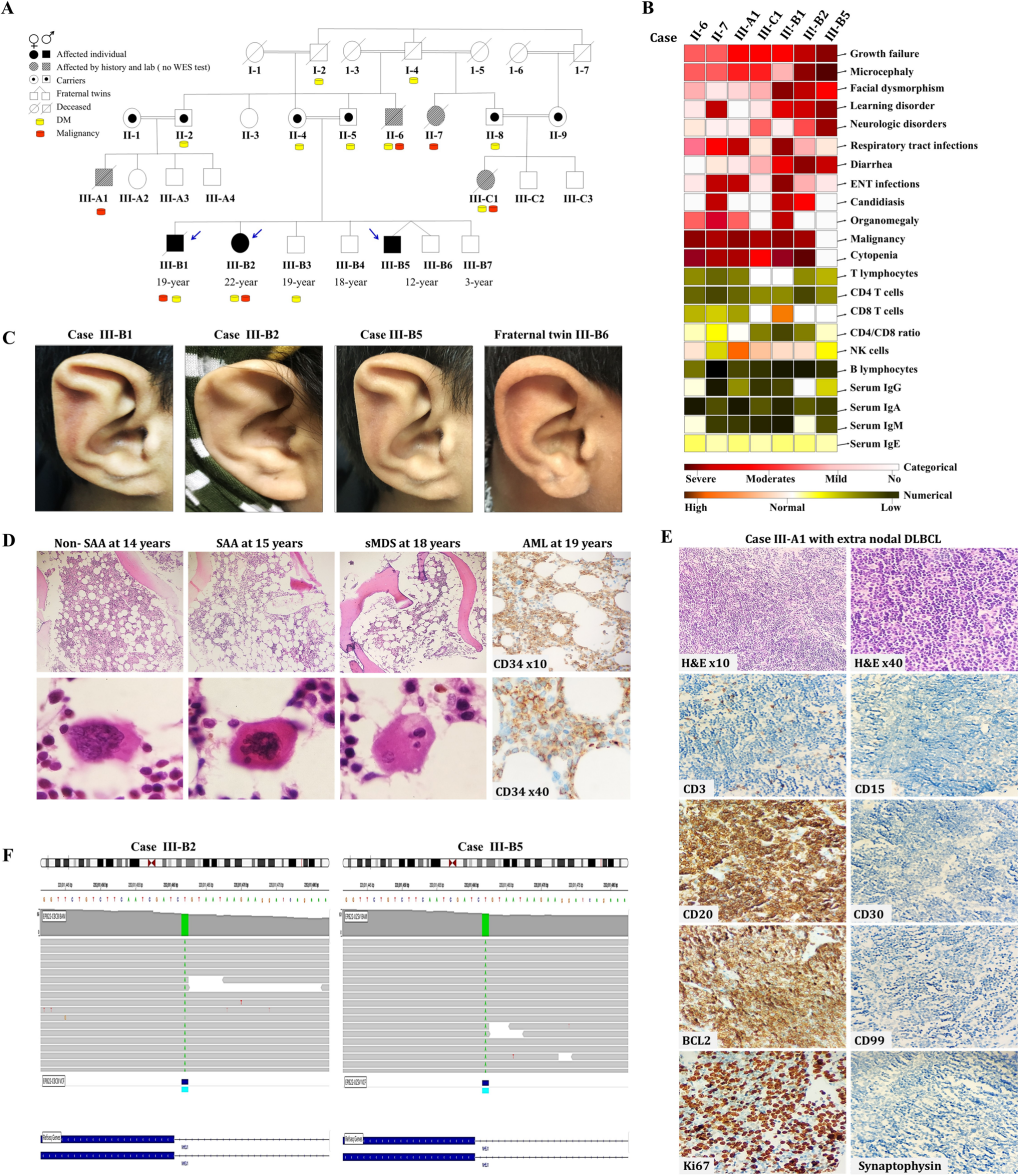
Phenotypic heterogeneity of *NHEJ1* genetic variants in family 1 with different types of hematological malignancies susceptibility. **A** Pedigree, clinical features, and molecular findings of *NHEJ1* mutation in homozygous and heterozygous individuals. In the pedigree, the 3 siblings Case III-B1, Case III-B2, and Case III-B5 are marked with blue arrows, respectively. Ages at time of manuscript writing are shown. **B** Representative matrix showed phenotypic heterogeneity among the affected family members. **C** Auricles anomalies; only affected member had an extra cartilage fold in the scapha portion of the ear involved both ears "Stahl’s ear". **D** Representative bone marrow core biopsies result from Case III-B1 (patient 1) revealed progression of hypoplastic bone marrow (BM) during disease course (BM hypocellularity 40%, 25%, 10–5%, respectively). At the age of 14, biopsy showed hypocellular BM without evidence of cellular dysplasia, malignancy, fibrosis, or granulomas with normal megakaryocytes that progressed to severe hypoplastic BM at the age of 15, then secondary MDS with multilineage dysplasia at the age of 18, which was complicated with AML at age of 19. **E** Representative soft tissue right para spinal mass biopsy result from Case III-A1 revealed diffuse large B-cell lymphoma (DLBCL). **F** Representative integrative genomics viewer graphics for visualization of homozygous *NHEJ1* variant that has been identified in DNA derived from blood sample of the Case III-B2 (patient 2) and her asymptomatic brother (III-B5) by whole exome sequencing. The green bar represents the mutations in a homozygous canonical splice site variant, c.530-2A > T, located in intron 4 of the *NHEJ1* gene (NM_ 024782.3). Abbreviations: AML: acute myeloid leukemia, CD: cluster of differentiation, DLBCL: diffuse large B-cell lymphoma, DM; Diabetes mellitus, ENT; ear, nose, and throat, Ig; immunoglobulin, NK; natural killer cells, SAA: severe aplastic anemia, sMDS: secondary myelodysplastic syndrome

In an Egyptian family (Fig. 2A), seven patients exhibited FHM with characteristic clinical features, including growth retardation, microcephaly, facial dysmorphism, and immunodeficiency (for detailed case reports, see the supplemental file). Recurrent infections caused by bacterial, viral, and fungal pathogens were observed in all seven patients, with varying degrees of severity. Figure 2B illustrates the diverse malformations and phenotypic variability among family members, including growth retardation, learning disorders, and microcephaly, particularly in third-generation patients. Interestingly, only affected members displayed an extra cartilage fold in the scapha portion of the ear, known as "Stahl’s ear," which was present in both ears (Fig. 2C). Additionally, heterogeneity in growth, severity of immunodeficiency, degree of BMF, and progression to sMDS transformation were observed (Table 2, Figs. S4-5).

The proband (P1/III-B1) was diagnosed with immune thrombocytopenia (ITP) and SCID at age 11, non-severe AA at 14, which progressed to severe AA without karyotype abnormalities at 15, then to sMDS at 18, and ultimately to AML at 19 (Fig. 2D). His sister (P2/III-B2) was diagnosed with ITP at age 5, followed by SCID and severe hypoplastic BM without dysmorphic features at 18, progressing to sMDS with multilineage dysplasia (sMDS-MLD) at 22. Their uncle (P3/II-6) developed BMF at 18, followed by diffuse large B-cell lymphoma (DLBCL) at 23, while their aunt (P6/II-7) had BMF at 17, progressing to AML at 21. Two cousins (P4/III-A1 and P5/III-C1) developed DLBCL (Fig. 2E) and MDS with excess blasts (RAEB-2), respectively, before age 20, both preceded by BMF. Notably, the two patients who developed DLBCL experienced a higher frequency of recurrent viral infections compared to others, and one (III-C1) had an acute Epstein-Barr virus (EBV) infection prior to lymphoma diagnosis. Family history revealed consanguineous marriages and a range of hematologic and endocrine disorders, with the paternal grandfather dying of lung tumor complications at 72. Screening identified SCID in another symptomatic family member (III-B5). These findings highlight a complex interplay of genetic factors predisposing the family to various hematologic and endocrine disorders (Table 3).

To identify the genetic defects predisposed to SCID, BMF and leukemia/lymphoma in this family, WES was performed. The analysis revealed a homozygous canonical splice site variant, c.530-2A > T, in intron 4 of the *NHEJ1* gene (NM_ 024782.3) (Fig. 2F); based on the American College of Medical Genetics (ACMG) guidelines this variant was classified as likely pathogenic, leading to the diagnosis of Cernunnos deficiency.

##### Immunological landscape of *NHEJ1* variants

The impact of homozygous and heterozygous *NHEJ1* variants on the immune system was evaluated. Affected individuals exhibited B-cell cytopenia, reduced absolute counts of T-helper and total T cells, elevated natural killer (NK) cell percentages, and a T–B–NK + SCID phenotype. Serum levels of IgA, IgE, IgG, and IgM were significantly lower in affected individuals compared to carriers and healthy controls. No paroxysmal nocturnal hemoglobinuria (PNH) clones were detected in homozygous *NHEJ1* patients. Additionally, a strong association with type 1 diabetes mellitus (DM) was noted, and immune thrombocytopenia (ITP) frequently presented as the first hematological manifestation in affected individuals (Table 2).

### Malignancy profiles across patients with faulty DSBR: LIG4, NBS, and NHEJ1 insights

**Fig. 3:**
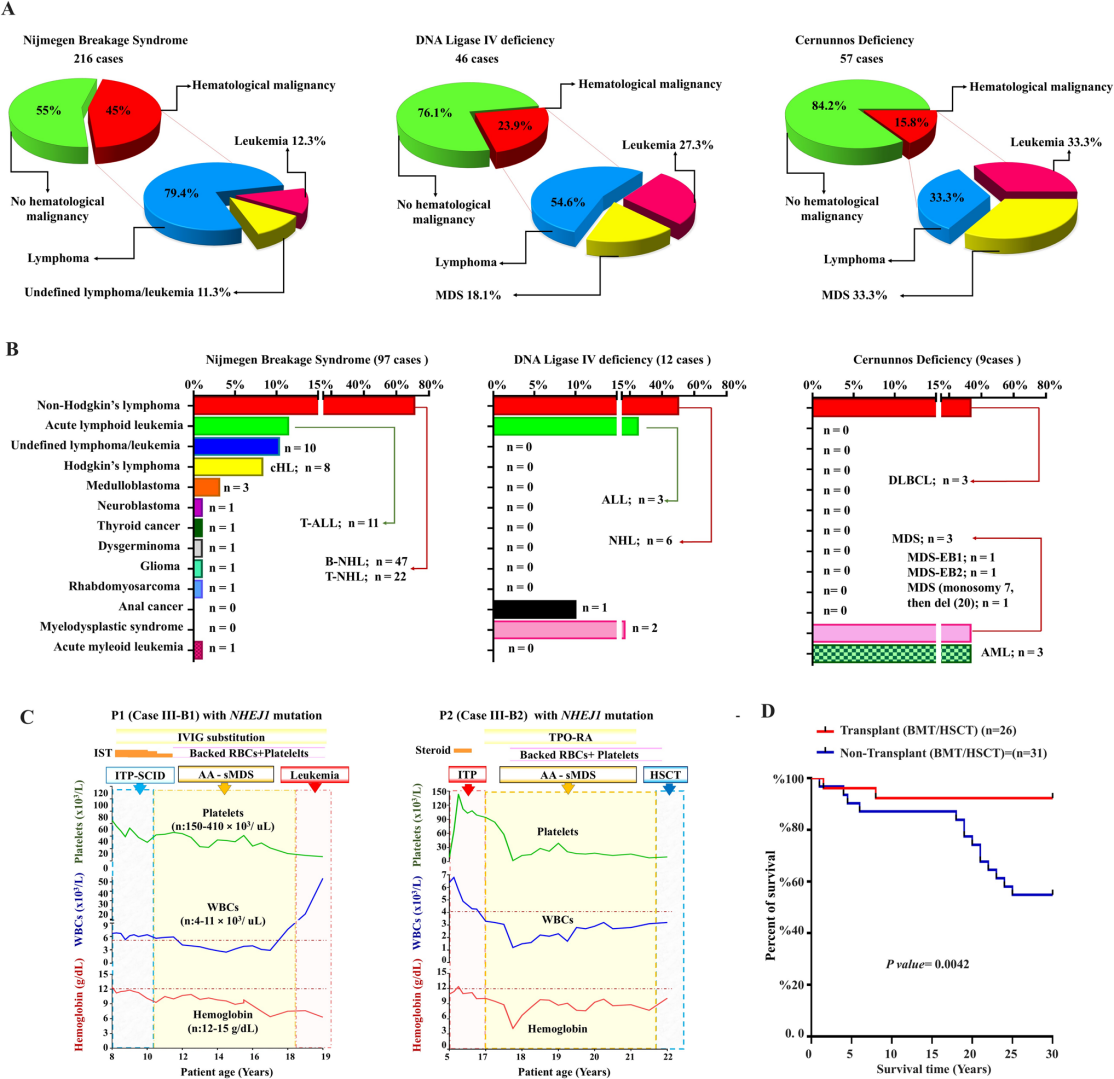
**A** and **B** Comparison of the percentage and types of hematological malignancies between patients with faulty double-strand break repair (NBS: Nijmegen breakage syndrome, Ligase IV syndrome, and non-homologous end joining deficiency). **C** The hematological parameters including white blood cell (WBC) count, hemoglobin level, and platelet count were monitored throughout the treatment and follow-up periods for two affected siblings (Case III-B1 & Case III-B2) with NHEJ1 deficiency. Initial measurements were taken prior to treatment initiation. Case III-B1 was slated for transplantation, but plans were postponed due to the unavailability of suitable non-carrier sibling donors or unrelated donors, resulting in unfortunate leukemia-related complications leading to the patient’s demise. However, his sister (Case III-B2), who was a candidate for matched sibling peripheral blood stem cell transplantation (PBSCT) from her brother (III-B6), exhibited a partial increase in platelet count after receiving thrombopoietin receptor agonist (TPO-RA) therapy. **D** A Kaplan–Meier curve illustrated that patients undergoing hematopoietic stem cell transplantation following a reduced-intensity conditioning regimen exhibited significantly improved survival rates. Normal ranges such as upper and lower limits are depicted in dashed lines. Normal ranges were considered according to the following: WBCs 4–11 × 10^3^/ uL, hemoglobin 12–15 g/dL, platelets 150–410 × 10^3^/uL. Abbreviations: ALL; acute lymphoblastic leukemia, BMT; bone marrow transplantation, cHL; classical Hodgkin lymphoma, Cy-A; cyclosporine-A, DLBCL; diffuse large B cell lymphoma, hMDS: hypoplastic myelodysplastic syndrome, HSCT: hematopoietic stem cell transplantation. MDS; myelodysplastic syndrome NHL: Non-Hodgkin lymphoma, sMDS; secondary myelodysplastic syndrome, TPO-RA: thrombopoietin receptor agonists, WBCs: white blood cells

A comparative analysis of 319 patients with DSBR disorders reported in the literature, including 46 with LIG4 deficiency, 216 with NBS, and 57 with autosomal recessive *NHEJ1* variants (seven from this study), revealed that malignancy poses a significant mortality risk for NBS patients. Over 45% of these individuals developed cancer, primarily of lymphoid origin, including diffuse DLBCL, T-ALL, unspecified lymphoma/leukemia, and Hodgkin’s lymphoma. Solid tumors such as medulloblastoma and rhabdomyosarcoma were also documented. Hematopoietic cancers accounted for 23.9% of cases in patients with LIG4 deficiency, particularly lymphomas (both EBV-positive and EBV-negative), lymphoid leukemias, and MDS. In contrast, hematologic malignancies (HM) represented 15.8% of cases in NHEJ1-deficient patients, with a tendency toward MDS/AML (Fig. 3A and B).

### Life-saving screening and tailored treatment approaches could delay clonal evolution and decrease therapy related mortality among patients with FHM

To prevent further clonal evolution and disease progression, comprehensive screening was conducted for all family members of patients with FHM. This included frequent BM morphological and cytogenetic evaluations, along with aggressive antimicrobial therapy while avoiding DNA-damaging agents. Patients with DNA-RD who developed MDS/AML were recommended to undergo a reduced-intensity conditioning (RIC) regimen prior to hematopoietic stem cell transplantation (HSCT). HSCT was also advised for patients with severe BMF or severe isolated cytopenia requiring transfusion support, poor-risk chromosome abnormalities, or significant dysplasia.

During the 6-year follow-up period of Family 1, surviving patients maintained stable overall health, with minimized radiographic imaging (X-ray and CT). Some cases showed improved blood counts, particularly patients 2, 9, 10, and 21, who were treated with thrombopoietin receptor agonists and/or danazol, while effective infection control was sustained until transplantation. However, proband III-B5, initially asymptomatic until age 10, was later diagnosed with SCID during family screening. He experienced recurrent infections and was diagnosed with attention deficit hyperactivity disorder (ADHD) by age 11, without progressing to BMF.

As of manuscript writing, the mortality rate was 33.3%, notably higher (43.8%) among patients with DNA-RD. This was attributed to disease progression or the lack of HLA-matched donors for HSCT. Individuals who did not undergo HLA-matched HSCT exhibited a significantly higher mortality rate. Conversely, patients who underwent HSCT following an RIC regimen showed markedly improved survival rates (Fig. 3C and D).

## Discussion

During a six-year study period, we screened 780 patients with HM for FHM, identifying 33 cases with clinical and laboratory features consistent with FHM from 19 families. Additionally, we conducted a comparative analysis involving 57 patients with NHEJ1 deficiency, 46 patients with LIG4 deficiency, and 216 patients with NBS. This study represents the first investigation into the genetic defects underlying FHM in the Egyptian population. These findings highlight the significance of comprehensive genetic assessment and familial screening to identify at-risk individuals, inform preventive strategies, and provide crucial insights for life-saving management. Firstly, the emergence of HM among patients with DNA-RD underscores the critical role of genomic stability maintenance in preventing oncogenic transformations. Our findings reveal that patients with DNA-RD, particularly those who presented initially with BMF and progressed to sMDS, have an increased predisposition to develop HM, such as leukemia, and lymphoma. This observation prompts further investigation into the underlying mechanisms connecting these seemingly disparate conditions. Notably, previous cross-sectional studies have reported incidences of MDS in FA ranging from 11 to 34% [27–29]. Additionally, the cumulative incidence of AML ranges from 10 to 37% by the age of 50, often manifesting during teenage years or early adulthood. Myelodysplastic syndrome and AML in FA are frequently, though not universally, preceded by BMF [27, 30]. While AML can manifest as a de novo condition in FA, our study reveals that AML commonly evolves from a preceding phase of MDS, characterized by a gradual increase in blast cell population over several months or years, not only in FA patients but also in those with NHEJ1 deficiency and DC. While previous publications have documented only three cases out of 50 patients associating malignancies like NHL, MDS, and AML, with NHEJ1 deficiency [31–33], our study presents new evidence. We report six new cases from a single family who have developed NHL, MDS, or AML, all preceded by BMF and sMDS. This sheds new light on the potential impact of NHEJ1 deficiency on predisposition to HM.

Secondary, MDS arises as a consequence of prior exposure to genotoxic insults, such as chemotherapy, radiation therapy, or environmental carcinogens, which exacerbate underlying DNA repair deficiencies, leading to clonal expansion and malignant transformation of hematopoietic progenitor cells [34]. The occurrence of sMDS in our cohort underscores the vulnerability of DNA repair-deficient cells to genotoxic stress and highlights the intricate interplay between exogenous exposures and endogenous DNA repair mechanisms in shaping disease progression. There are many suggested mechanisms linking these variants to impaired hematopoiesis, clonal evolution, and malignant transformation. Persistent DNA lesions, including double-strand breaks (DSBs) and replication stress, activate cell cycle checkpoints and apoptotic pathways, leading to impaired self-renewal capacity, stem cell exhaustion, and depletion of the hematopoietic stem cell pool. Another mechanism is the DNA repair deficiencies, can perturb the dynamic interactions between HSPCs and their niches, disrupting essential signaling pathways, such as Wnt, Notch, and TGF-β, involved in HSC maintenance, quiescence, and lineage specification [35]. Dysregulated niche interactions, characterized by altered cytokine production, inflammatory signaling, and niche remodeling, compromise the regenerative capacity and functional integrity of HSPCs, exacerbating hematopoietic dysfunction and disease progression in DNA-RD.

In our family with a variant in *NHEJ1* gene, the observed phenotypic heterogeneity can manifest across generations and among affected individuals, presenting a fascinating yet challenging aspect of clinical genetics diagnosis and management. Within the same family, individuals may exhibit varying degrees of disease severity, ranging from asymptomatic carriers to those with overt clinical manifestations, such as SCID, BMF, hMDS, or HM and familial cancer predisposition. Although numerous causative variants have been documented in individuals with Cernunnos deficiency, there exists no clear correlation between the type or location of the variant and the clinical manifestations or severity observed in affected patients (Table S2). In addition, while our patients harboring the same affected nucleotide, they presented with heterogeneous phenotypes and different clonal evolution. Several factors contribute to phenotypic variability within families with variants in *NHEJ1* gene. Genetic variants outside the *NHEJ1* locus may influence disease expression and modify the phenotypic spectrum observed within a family. Furthermore, environmental factors, including exposure to infectious agents, toxins, or radiation, may interact with genetic predispositions and modulate disease expression. Variations in environmental exposures among family members can contribute to differences in disease penetrance and expressivity. These can lead to divergent clinical outcomes, contributing to phenotypic heterogeneity, different clonal evolution and lymphoma susceptibility as occurred in the two patients with DLBCL who had EBV or repeated viral infection. Additionally, variations in the distribution and extent of somatic mosaicism may lead to differences in disease severity and clinical features among affected individuals. Finally, stochastic events occurring during embryonic development or throughout life, such as spontaneous mutations or epigenetic changes, can also contribute to phenotypic heterogeneity within families.

The recognition of phenotypic variability within families with variants in the *NHEJ1* gene has important implications for screening recommendations, genetic counseling, individualized clinical management strategies tailored to the specific phenotypic manifestations, and advancing research efforts aimed at improving patient outcomes.

Consistent with our findings, a study involving patients with DSBR identified 77 individuals affected by LIG4 deficiency, Cernunnos deficiency, or NBS. Among them, 73 patients underwent an RIC regimen before HSCT, achieving a significantly higher survival rate (69%) [36]. This underscores the importance of carefully considering the specific diagnosis and indication, the timing of the procedure, and, perhaps most crucially, the balance of risks between HSCT and disease progression when making decisions regarding HSCT.

In contrast, our patients without DNA-RD exhibit a spectrum of hematological malignancies, including MPNs, acute leukemia, and lymphoma, as well as solid malignancies, which may reflect distinct pathophysiological mechanisms driving malignant transformation. Dysplastic conditions such as DC are characterized by ineffective hematopoiesis and dysregulated differentiation, whereas MPNs are characterized by clonal proliferation of hematopoietic stem cells. While the precise etiology of these conditions remains incompletely understood, the presence of familial predispositions implicates genetic factors in disease pathogenesis, with potential contributions from inherited genetic variants or shared environmental exposures within affected families.

Familial chronic MPNs are defined when at least two relatives in the same pedigree have MPNs [37]. Genetic variants of MPN drivers, such as *JAK2*, *CALR*, and *MPL*, are acquired somatically, even in familial cases. This suggests that there might be a hereditary predisposition to acquiring one of these MPN driver variants, even though the specific germline variants responsible for familial MPN are largely unidentified [37]. Olcaydu et al. examined the role of the *JAK2* GGCC haplotype in familial MPNs and found no significant difference in haplotype frequency or *JAK2* mutation risk between familial and sporadic cases. Approximately 30% of familial MPN patients lack the GGCC haplotype, and familial cases show higher penetrance than the haplotype alone. No germline mutations in *TET2, CBL*, or *MPL* were found to co-segregate with the disease [38]. These findings align with earlier studies that excluded linkage to the *JAK2* gene locus based on microsatellite marker analysis in four familial PV pedigrees [39]. These findings suggest the GGCC haplotype contributes to *JAK2* mutation susceptibility but does not fully explain familial clustering. Recent genome-wide association studies identified the *ATM L2307F* variant (in ~ 8% of familial cases) and other mutations in DNA repair and telomere regulation genes (*CHEK2, FANCD2, RET, RTEL1*) in ~ 7 to 3% of familial cases [15, 16]. However, understanding of the germline defect underlying familial MPN remains limited.

Limited studies of familial clusters of MPNs have been reported; however, variants of the *JAK2* or *CALR* gene have been reported in the same pedigree in many studies [40–42], suggesting that genetic predisposition to the acquisition of these variants is supposed to be inherited. In addition, our patients with familial MPNs develop the same type of complications (thrombosis) as previously described [41]. The inheritance pattern of familial MPNs is consistent with an autosomal dominant trait with decreased penetrance. Intriguingly, among *JAK2*V617F mutated patients, one patient developed renal cell carcinoma (RCC) after years of ET diagnosis. This coexistence was reported previously [43], as JAK2 activation is recognized in RCC [44]. Another patient with PV and *JAK2 V617F* variant has β-thalassemia minor with a strong family history of β-thalassemia, gastric cancer, and lymphoma. This observation was confirmed by many studies that showed the prevalence *JAK2 V617F* variant (22.2%) in β-thalassemia major patients [45] or heterozygous β-thalassemia [46]. Also, PV patients developed gastric cancer [47]. It can be postulated that “stress erythropoiesis”, known to occur in patients with thalassemia, increases the probability of somatic variant in *JAK2,* leading to the development of PV. We hypothesize that genetic lesions responsible for the familial clustering of MPNs are not confined to myeloid hematopoietic cells but instead lead to an overall increase in carcinogenesis.

Additionally, we identified *BRCA2* variants in a family with two siblings (age 41 and 44 years) of multiple myeloma (MM) and five cases of breast cancer. Evidence of an increased risk of MM in relatives of carriers of *BRCA1* or *BRCA2* variants has been reported [48]. Also, familial MM was reported to bear a nonsense variant in exon 27 of *BRCA2* that corresponds to a Lys 3326 Stop substitution predicted to cause the loss of the final 93 amino acids of the BRCA2 protein [49]. A recent study analyzed germline exomes from two MM cohorts (895 and 786 individuals) revealed that MM patients are more likely than (134,187) healthy individuals to carry pathogenic germline variants (PGVs) in *BRCA1* (OR = 3.9) and *BRCA2* (OR = 7.0) genes, which significantly increase cancer risk. While the disease primarily affects older adults over 50, patients with these genetic variants tend to be diagnosed at a younger age and often have a personal or family history of cancer [50]

Moreover, the consistent presence of family histories of both hematological malignancies and solid tumors across all cases underscores the potential genetic and environmental factors contributing to disease predisposition. This study has several limitations. The small sample size of 33 confirmed cases may limit the generalizability of the findings. The reliance on gene panel testing and WES could miss certain genetic variants, such as those in non-coding regions or structural variants. Additionally, the study did not extensively explore environmental factors and their interaction with genetic predispositions, which could provide a more comprehensive understanding of disease progression. Future collaborative efforts across multiple centers including diverse populations will be crucial to validate these findings and enhance our understanding of FHM.

This study underscores the importance of interdisciplinary collaboration and genetic profiling in elucidating the underlying mechanisms and familial associations of HM. Our study delves into the implications of identifying variants for life-saving screening and prevention measures for at-risk family members as well as for diagnosis and tailored treatment approaches based on genetic profiles in patients with a family history of HM.

## Supplementary Information

Below is the link to the electronic supplementary material.

ESM 1 (PDF 2.19 MB)

## Data Availability

Data is provided within the manuscript or supplementary information files.

## References

[1] Churpek JE, Lorenz R, Nedumgottil S, Onel K, Olopade OI, Sorrell A, Owen CJ, Bertuch AA, Godley LA (2013) Proposal for the clinical detection and management of patients and their family members with familial myelodysplastic syndrome/acute leukemia predisposition syndromes. Leuk Lymphoma 54(1):28–35 22691122 10.3109/10428194.2012.701738

[2] Rönkkö R, Hirvonen E, Malila N (2021) Familial aggregation of early-onset haematological malignancies 193(6):1134–1141 10.1111/bjh.17477 34002362

[3] Satgé D, Seidel MG (2018) The pattern of malignancies in Down syndrome and its potential context with the immune system. Front Immunol 9:3058 30631328 10.3389/fimmu.2018.03058 PMC6315194

[4] Tawana K, Brown AL, Churpek JE (2022) Integrating germline variant assessment into routine clinical practice for myelodysplastic syndrome and acute myeloid leukaemia: current strategies and challenges. Br J Haematol 196(6):1293–1310 34658019 10.1111/bjh.17855

[5] DiNardo CD, Bannon SA, Routbort M, Franklin A, Mork M, Armanios M, Mace EM, Orange JS, Jeff-Eke M, Churpek JE et al (2016) Evaluation of patients and families with concern for predispositions to hematologic malignancies within the hereditary hematologic malignancy clinic (HHMC). Clin Lymphoma Myeloma Leuk 16(7):417–428.e412 27210295 10.1016/j.clml.2016.04.001 PMC4925265

[6] Garber JE, Offit K (2005) Hereditary cancer predisposition syndromes. J Clin Oncol 23(2):276–292 15637391 10.1200/JCO.2005.10.042

[7] Tischkowitz M, Dokal I (2004) Fanconi anaemia and leukaemia – clinical and molecular aspects. Br J Haematol 126(2):176–191 15238138 10.1111/j.1365-2141.2004.05023.x

[8] Jones CH, Pepper C, Baird DM (2012) Telomere dysfunction and its role in haematological cancer. Br J Haematol 156(5):573–587 22233151 10.1111/j.1365-2141.2011.09022.x

[9] Moshous D, Pannetier C, de Chasseval R, le Deist F, Cavazzana-Calvo M, Romana S, Macintyre E, Canioni D, Brousse N, Fischer A (2003) Partial T and B lymphocyte immunodeficiency and predisposition to lymphoma in patients with hypomorphic mutations in Artemis. J Clin Investig 111(3):381–387 12569164 10.1172/JCI16774 PMC151863

[10] Rollinson S, Kesby H, Morgan GJ (2006) Haplotypic variation in MRE11, RAD50 and NBS1 and risk of non-Hodgkin’s lymphoma. Leuk Lymphoma 47(12):2567–2583 17169801 10.1080/10428190600909743

[11] Bohgaki T, Bohgaki M, Hakem R (2010) DNA double-strand break signaling and human disorders. Genome integrity 1:1–14 21054854 10.1186/2041-9414-1-15 PMC2993650

[12] Roddam PL, Allan JM, Dring AM, Worrillow LJ, Davies FE, Morgan GJ (2010) Non-homologous end-joining gene profiling reveals distinct expression patterns associated with lymphoma and multiple myeloma. Br J Haematol 149(2):258–262 20148879 10.1111/j.1365-2141.2010.08088.x

[13] Rumi E, Cazzola M (2017) Diagnosis, risk stratification, and response evaluation in classical myeloproliferative neoplasms. Blood 129(6):680–692 28028026 10.1182/blood-2016-10-695957 PMC5335805

[14] Harutyunyan AS, Giambruno R, Krendl C, Stukalov A, Klampfl T, Berg T, Chen D, Milosevic Feenstra JD, Jäger R, Gisslinger B (2016) Germline RBBP6 mutations in familial myeloproliferative neoplasms. Blood, The Journal of the American Society of Hematology 127(3):362–365 10.1182/blood-2015-09-668673 PMC5043418 26574608

[15] Di Marino L, Ramundo F, Malara T, Rossi M, Maggi R, Fosso F, Betti S, Rossi E, Leone G, Sica S (2024) Familial predisposition in myeloproliferative neoplasms: an NGS-driven analysis. Blood 144:1763

[16] Braunstein EM, Imada E, Pasca S, Wang S, Chen H, Alba C, Hupalo DN, Wilkerson M, Dalgard CL, Ghannam J (2023) Recurrent germline variant in ATM associated with familial myeloproliferative neoplasms. Leukemia 37(3):627–635 36543879 10.1038/s41375-022-01797-6

[17] Sharma R, Lewis S, Wlodarski MW (2020) DNA repair syndromes and cancer: insights into genetics and phenotype patterns. Front Pediatr 8:570084 33194896 10.3389/fped.2020.570084 PMC7644847

[18] Bluteau O, Sebert M, Leblanc T, Peffault de Latour R, Quentin S, Lainey E, Hernandez L, Dalle J-H, Sicre de Fontbrune F, Lengline E (2018) A landscape of germ line mutations in a cohort of inherited bone marrow failure patients. Blood, J Am Soc Hematol 131(7):717–732 10.1182/blood-2017-09-806489 29146883

[19] Arber D, Orazi A, Hasserjian R, Brunning R, Le Beau M, Porwit A, Tefferi A, Levine R, Bloomfield C, Cazzola M (2017) Introduction and overview of the classification of myeloid neoplasms. WHO Classification of Tumours Lyon. France: IARC, pp 16–27

[20] Bennett JM, Orazi A (2009) Diagnostic criteria to distinguish hypocellular acute myeloid leukemia from hypocellular myelodysplastic syndromes and aplastic anemia: recommendations for a standardized approach. Haematologica 94(2):264 19144661 10.3324/haematol.13755 PMC2635414

[21] Attygalle AD, Chan JK, Coupland SE, Du M-Q, Ferry JA, Jong Dd, Gratzinger D, Lim MS, Naresh KN, Nicolae A (2024) The 5th edition of the world health organization classification of mature lymphoid and stromal tumors–an overview and update. Leukemia & Lymphoma , pp 1–17 10.1080/10428194.2023.2297939 38189838

[22] Khoury JD, Solary E, Abla O, Akkari Y, Alaggio R, Apperley JF, Bejar R, Berti E, Busque L, Chan JK (2022) The 5th edition of the World Health Organization classification of haematolymphoid tumours: myeloid and histiocytic/dendritic neoplasms. Leukemia 36(7): 1703–1719 10.1038/s41375-022-01613-1 PMC9252913 35732831

[23] der Auwera V (2013) From FastQ data to high confidence variant calls: the genome analysis toolkit best practices pipeline. Curr Protoc Bioinformatics 43:1 10.1002/0471250953.bi1110s43 PMC4243306 25431634

[24] Seo GH, Kim T, Choi IH, Jy Park, Lee J, Kim S, Won Dg OhA, Lee Y, Choi J (2020) Diagnostic yield and clinical utility of whole exome sequencing using an automated variant prioritization system. EVIDENCE Clinical genetics 98(6):562–570 32901917 10.1111/cge.13848 PMC7756481

[25] Richards S, Aziz N, Bale S, Bick D, Das S, Gastier-Foster J, Grody WW, Hegde M, Lyon E, Spector E (2015) Standards and guidelines for the interpretation of sequence variants: a joint consensus recommendation of the American College of Medical Genetics and Genomics and the Association for Molecular Pathology. Genet Med 17(5):405–423 25741868 10.1038/gim.2015.30 PMC4544753

[26] Elbadry MI, Tawfeek A, Hirano T, El-Mokhtar MA, Kenawey M, Helmy AM, Ogawa S, Mughal MZ, Nannya Y (2024) A rare homozygous variant in TERT gene causing variable bone marrow failure, fragility fractures, rib anomalies and extremely short telomere lengths with high serum IgE. Br J Haematol 204(3):1086–1095 37926112 10.1111/bjh.19176

[27] Butturini A, Gale RP, Verlander PC, Adler-Brecher B, Gillio AP, Auerbach AD (1994) Hematologic abnormalities in fanconi anemia: an international fanconi anemia registry study [see comments] 8068955

[28] Schaison G, Leverger G, Yildiz C, Berger R, Bernheim A, Gluckman E (1983) Fanconi’s anemia. Incidence of its development into leukemia. Presse Medicale (Paris, France: 1983) 12(20):1269–1274 6222298

[29] Rackoff WR, Orazi A, Robinson CA, Cooper RJ, Alter BP, Freedman MH, Harris RE, Williams DA (1996) Prolonged administration of granulocyte colony-stimulating factor (filgrastim) to patients with fanconi anemia: a pilot study 8781414

[30] Rosenberg PS, Alter BP, Ebell W (2008) Cancer risks in fanconi anemia: findings from the German fanconi anemia registry. Haematologica 93(4):511–517 18322251 10.3324/haematol.12234

[31] Patiroglu T, Akar HH, van der Burg M, Kontas O (2015) A case of XLF deficiency presented with diffuse large B cell lymphoma in the brain. Clinical immunology (Orlando, Fla) 161(2):394–395 26119972 10.1016/j.clim.2015.06.009

[32] Kager L, Jimenez Heredia R, Hirschmugl T, Dmytrus J, Krolo A, Müller H, Bock C, Zeitlhofer P, Dworzak M, Mann G et al (2018) Targeted mutation screening of 292 candidate genes in 38 children with inborn haematological cytopenias efficiently identifies novel disease-causing mutations 182(2):251–258 10.1111/bjh.15389 PMC6079646 29797310

[33] Arunachalam AK, Maddali M, Aboobacker FN, Korula A, George B, Mathews V, Edison ES (2021) Primary Immunodeficiencies in India: Molecular Diagnosis and the Role of Next-Generation Sequencing 41(2):393–413 10.1007/s10875-020-00923-2 PMC7610931 33225392

[34] Calvete O, Mestre J (2023) The secondary myelodysplastic neoplasms (MDS) Jigsaw 15(5) 10.3390/cancers15051483 PMC10000488 36900275

[35] Mendelson A, Frenette PS (2014) Hematopoietic stem cell niche maintenance during homeostasis and regeneration. Nat Med 20(8):833–846 25100529 10.1038/nm.3647 PMC4459580

[36] Slack J, Albert MH, Balashov D, Belohradsky BH, Bertaina A, Bleesing J, Booth C, Buechner J, Buckley RH, Ouachée-Chardin M (2018) Outcome of hematopoietic cell transplantation for DNA double-strand break repair disorders. J Allergy Clin Immunol 141(1):322–328.e310 28392333 10.1016/j.jaci.2017.02.036 PMC5632132

[37] Rumi E, Cazzola M (2017) Advances in understanding the pathogenesis of familial myeloproliferative neoplasms. Br J Haematol 178(5):689–698 28444727 10.1111/bjh.14713

[38] Olcaydu D, Rumi E, Harutyunyan A, Passamonti F, Pietra D, Pascutto C, Berg T, Jäger R, Hammond E, Cazzola M et al (2011) The role of the JAK2 GGCC haplotype and the TET2 gene in familial myeloproliferative neoplasms. Haematologica 96(3):367–374 21173100 10.3324/haematol.2010.034488 PMC3046267

[39] Kralovics R, Stockton DW, Prchal JT (2003) Clonal hematopoiesis in familial polycythemia vera suggests the involvement of multiple mutational events in the early pathogenesis of the disease. Blood 102(10):3793–3796 12829587 10.1182/blood-2003-03-0885

[40] Alati C, Martino B, Marino A, Ronco F, Priolo M, Nobile F (2010) Familial chronic myeloproliferative neoplasms. American Society of Hematology, In.

[41] Rumi E (2008) Familial chronic myeloproliferative disorders: the state of the art. Hematol Oncol 26(3):131–138 18484677 10.1002/hon.863

[42] Maffioli M, Genoni A, Caramazza D, Mora B, Bussini A, Merli M, Giorgino T, Casalone R, Passamonti F (2014) Looking for CALR mutations in familial myeloproliferative neoplasms. Leukemia 28(6):1357–1360 24441291 10.1038/leu.2014.33

[43] Namdaroğlu S, Tekgündüz E, Altuntaş F (2016) Coexistence of essential thrombocythemia, iron-refractory iron deficiency anemia and renal cell carcinoma. Hematol Rep 8(1):6235 27103977 10.4081/hr.2016.6235 PMC4815946

[44] Gupta S, Cheville JC, Jungbluth AA, Zhang Y, Zhang L, Chen Y-B, Tickoo SK, Fine SW, Gopalan A, Al-Ahmadie HA (2019) JAK2/PD-L1/PD-L2 (9p24.1) amplifications in renal cell carcinomas with sarcomatoid transformation: implications for clinical management. Mod Pathol 32(9):1344–1358 30996253 10.1038/s41379-019-0269-x PMC7189735

[45] Krichevsky S, Borohovitz A, Prus J, Perlman R, Weinberg I, Abramowitz J, Treves A, Fibach E, Ben-Yehuda D (2010) The JAK2V617F mutation in non-MPD Individuals occurs at a low frequency during erythroid differentiation and not in the stem cells. Blood 116(21):1583

[46] Kottas K, Marathonitis A, Nodarou A, Kanellis G, Christopoulos K, Christopoulos C (2020) Polycythemia vera in a patient with heterozygous beta-thalassemia: coincidence or causal relationship? Cureus 12(11) 10.7759/cureus.11589 PMC7749832 33364111

[47] Ayvaz O, Yavasoglu I, Kadikoylu G, Meydan N, Barutca S, Bolaman Z (2010) The development of gastric cancer in a patient with polycythemia Vera, 3P deletion, and JAK2 V617F mutation. J Gastrointest Cancer 41(4):254–256 20306156 10.1007/s12029-010-9142-3

[48] Struewing JP, Hartge P, Wacholder S, Baker SM, Berlin M, McAdams M, Timmerman MM, Brody LC, Tucker MA (1997) The risk of cancer associated with specific mutations of BRCA1 and BRCA2 among Ashkenazi Jews. N Engl J Med 336(20):1401–1408 9145676 10.1056/NEJM199705153362001

[49] Lynch HT, Sanger WG, Pirruccello S, Quinn-Laquer B, Weisenburger DD (2001) Familial multiple myeloma: a family study and review of the literature. J Natl Cancer Inst 93(19):1479–1483 11584064 10.1093/jnci/93.19.1479

[50] Thibaud S, Subaran RL, Newman S, Lagana A, Melnekoff DT, Bodnar S, Ram M, Soens Z, Genthe W, Brander T (2024) Multiple myeloma risk and outcomes are associated with pathogenic germline variants in DNA repair genes. Blood Cancer Discovery 5(6):428–441 39283238 10.1158/2643-3230.BCD-23-0208 PMC11528192

