# Supplementary material for: Genetic variants in NHEJ1 and related DNA repair disorders: insights into phenotypic heterogeneity and links to hypoplastic myelodysplastic syndromes and familial hematological malignancies susceptibility
Source: Ann Hematol. 2025 Mar 6;104(3):1633–53. doi: 10.1007/s00277-025-06257-6 (PMC12031859; doi:10.1007/s00277-025-06257-6)
Supplement: Supplementary file 1 — (PDF 2.19 MB) [file 277_2025_6257_MOESM1_ESM.pdf]

Genetic variants in *NHEJ1* and Related DNA Repair Disorders: Insights into Phenotypic Heterogeneity and Links to Hypoplastic Myelodysplastic Syndromes and Familial Hematological Malignancies Susceptibility

Mahmoud I. Elbadry (1)*, Elsayed Abdelkreem (2), Ahmed Tawfeek (3), Go Hun Seo(4), Shereen Philip Aziz(3)

1) Department of Internal Medicine, Division of Haematology, Faculty of Medicine, Sohag University, Sohag, Egypt.
2) Department of Paediatrics, Faculty of Medicine, Sohag University
3) Department of Clinical and Chemical Pathology, Faculty of Medicine, Sohag University, Sohag, Egypt.
4) Medical genetics division, 3billion Inc., Seoul, South Korea

AUTHOR INFORMATION

1. **Mahmoud I. Elbadry** MD PhD
   - Department of Internal Medicine, Division of Haematology, Faculty of Medicine, Sohag University, Sohag, 82524, Egypt.
   - **Email:** mahmoudibrahem837@gmail.com, mahmoudibrahim@med.sohag.edu.eg
   - **ORCID ID:** https://orcid.org/0000-0001-5487-9493

2. **Elsayed Abdelkreem** MD PhD
   - Department of Paediatrics, Faculty of Medicine, Sohag University
   - **Email:** d.elsayedmohammed@med.sohag.edu.eg
   - **ORCID ID:** 0000-0002-8976-2989

3. **Ahmed Tawfeek** MD PhD
   - Department of Clinical and Chemical Pathology, Faculty of Medicine, Sohag University, Sohag, 82524, Egypt.
   - **Email:** Ahmed.tawfeek@med.sohag.edu.eg
   - **ORCID ID:** 0000-0003-2409-0517

4. **Go Hun Seo,** MD
   - Medical genetics division, 3billion Inc., Seoul, South Korea
   - **Email:** ghseo@3billion.io
   - **ORCID:** 0000-0003-1518-1791

5. **Shereen Philip Aziz** MD PhD
   - Department of Clinical and Chemical Pathology, Faculty of Medicine, Sohag
   - University, Sohag, 82524, Egypt.
   - **Email:** sanymn@yahoo.com
   - **ORCID ID:** 0009-0009-7952-3798

++Corresponding author:

Mahmoud I. Elbadry MD PhD

Address: Department of Internal Medicine, Division of Hematology, Faculty of Medicine, Sohag University, Nasr City, Eastern Avenue, University Street, Sohag, Egypt
E-mail: mahmoudibrahem837@gmail.com, mahmoudibrahim@med.sohag.edu.eg
Postal Code: 82524
Phone: +2-01065964083 **Fax:** +2-093-4609304
PATIENTS

SUPPLEMENTAL METHODS

LABORATORY INVESTIGATIONS

- **Routine hematological and biochemical investigations**
- **Autoimmune disorders screening**
- **Flow cytometry analysis**
- **Standard Cytogenetic Analysis**
- **Diepoxybutane chromosomal breakage**
- **DNA extraction**
- **Whole exome sequencing and data analysis**
- **Telomere length measurement**

RADIOLOGICAL ASSESSMENT

DUAL-ENERGY X-RAY ABSORPTIOMETRY

HISTOPATHOLOGICAL STUDIES

TREATMENT

FOLLOW-UP

STATISTICAL ANALYSES

SUPPLEMENTAL TABLES

Table S1. Summary of demographic, clinical, and molecular observations in individuals with Cernunnos deficiency
Table S2. Summary of demographic, and type of malignancy in 216 individuals with Nijmegen Breakage Syndrome
Table S4: Summary of demographic, and type of malignancy in 46 individuals with DNA Ligase IV deficiency

SUPPLEMENTAL FIGURES

Figure S1. . Flow chart of the study patients
Figure S2. . Diepoxybutane (DEB) induced chromosomal breakage assay.
Figure S3. . Pedigree, clinical features, malignancy type of family 3
Figure S4. . Growth curves of family's-1 cases
Figure S5. . Representative bone marrow core biopsies and peripheral blood

SUPPLEMENTAL REFERENCES

Figure S1. Flow diagram of patients referred to our hematology unit last 6 years

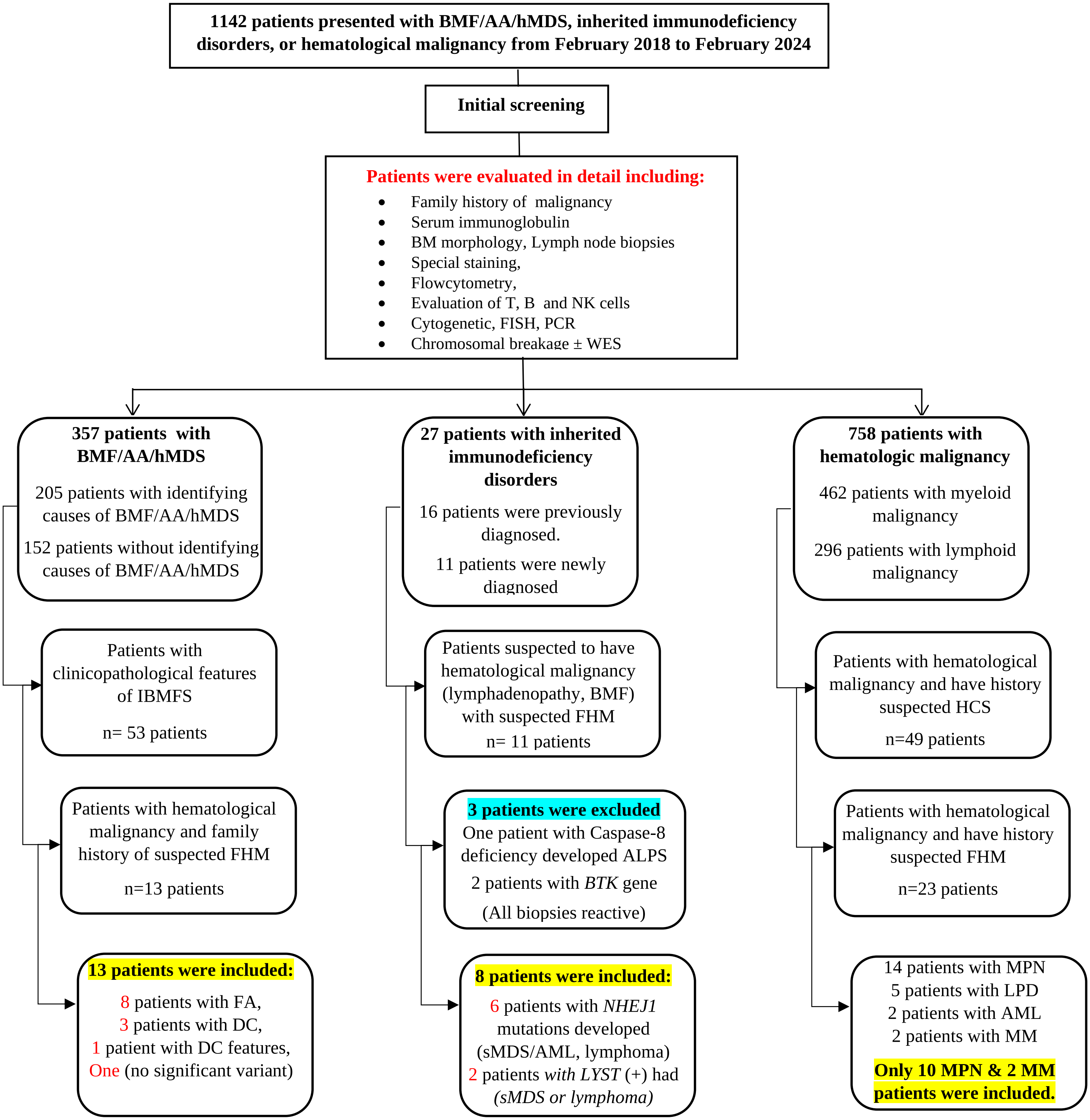

AA, aplastic anemia; ALPS, autoimmune lymphoproliferative syndrome; AML, acute myeloid leukemia; BMF, bone marrow failure; DC, Dyskeratosis congenita; FA, Fanconi anemia; FHM, familial hematological malignancy; HCS, hereditary cancer syndrome; hMDS, hypoplastic myelodysplastic syndrome; IBMFS, inherited bone marrow failure syndromes; LPD, lymphoproliferative disorders; MDS, myelodysplastic syndrome; MM, Multiple myeloma; MPN, myeloproliferative neoplasm; sMDS, secondary myelodysplastic syndromes

Diagnosis and clinical characteristic of the family 1 *NHEJ1*

Combined clinical and molecular data were carefully reviewed in each of the 3 patients of family 1, and the rest family members. Clinical characteristics of the individuals were reviewed, including physical abnormalities, growth, skeletal, neurological, genitourinary, cardiac, lung, liver, skin, nail, dermatological features or hair, in addition to the age of first fractures or hematological symptoms, cytopenias, bone marrow evaluation, family history of bone, hematological disorders, malignancy, and any other relevant information .

These 7 patients had criteria of severe combined immunodeficiency (SCID), growth failure, facial dysmorphism, microcephaly, learning disorders, BMFS, and predisposition to develop lymphoma and leukemia. This consanguineous Egyptian family was investigated for DNA Double-Strand Break Repair disorders due to their clinical history. At their first clinical evaluation at the Sohag University Hospital, a detailed clinical evaluation was recorded, and peripheral blood was collected for immunological, and genetic testing. As part of the clinical investigation for BMF and DNA Double-Strand Break Repair disorders, the probands were assessed for blood, lung, and liver diseases; they underwent BM biopsy, chest and abdomen ultrasound, and pulmonary function tests. After confirmation of molecular diagnosis, their parents, living siblings, and remaining family members were also screened for blood disorders and underwent ultrasound of the chest and abdomen. They refused BM biopsy due to financial reasons. Screened subjects denied tobacco use and alcohol consumption.

Diagnosis of acquired BMF patients with AA with and without PNH clones or hypoplastic MDS.

A total of 152 patients with hypocellular BM and suspected to have AA were assessed consecutively at the Department of Internal Medicine, Division of Hematology, Sohag University hospitals. Patients who were newly diagnosed with acquired AA (idiopathic) between 2018 and 2024 after BM aspirations, BM biopsies, cytogenetic with negative results of autoimmune antibodies, viral studies scan. These patients were enrolled in an observational comparison study to determine the difference in the clinical and hematological phenotypes between patients with acquired and inherited BMF. Also, Glycosylphosphatidylinositol-anchored protein (GPI-AP)− Gs for paroxysmal nocturnal hemoglobinuria (PNH) were performed as described previously [1]. Diagnosis of PNH/AA abnormal increase was defined as the presence of $CD55^{-}CD59^{-}CD235a^{+}$ RBCs, fluorescein labeled proaerolysin $(FLAER)^{-}CD11b^{+}$ or $CD55^{-}CD59^{-}CD11b^{+}$ granulocytes [1] using FACS Canto II (BD Biosciences, San Jose, USA). The results were analyzed with the FlowJo v10.1 software program (Tree star, Ashland, USA). The severity and response criteria were as previously described[2, 3].

Criteria of AA severity[3]

Severity	Criteria
**Severe aplastic anemia (SAA)**	Marrow cellularity <25% (or 25–50% with <30% residual haematopoietic cells), plus at least 2 of:<br>• Neutrophils <0.5 x 10^9/L,<br>• Platelets <20 x 10^9/L<br>• Reticulocyte count <20 x 10^9/L
**Very Severe AA (VSAA)**	As for SAA but neutrophils <0.2 x 10^9/L
**Non-severe AA (NSAA)**	AA not fulfilling the criteria for SAA or VSAA

Criteria for response to immunosuppressive therapy (IST) in AA[3]

**(A) Response criteria following IST in severe AA**	
**None**	Still fulfil severe disease criteria
**Partial**	- Transfusion independent - No longer meet criteria for severe disease
**Complete**	Hemoglobin concentration normal for age and gender<br>Neutrophil count >1·5 × 10^9/L<br>Platelet count >150 × 10^9/L
**(B) Response criteria following IST for non-severe AA**	
**None**	Blood counts are worse, or do not meet criteria below
**Partial**	Transfusion independence (if previously dependent)<br>*or* doubling or normalization of at least one cell line<br>*or* increase of baseline<br>• hemoglobin concentration of >30 g/L (if initially <60)<br>• neutrophils of >0.5 × 10^9/L (if initially <0·5)<br>• platelets of >20 × 10^9/L (if initially <20)
**Complete**	Same criteria as for severe disease

AA, aplastic anemia; IST, immunosuppressive therapy

Characteristic of acquired BMF patients with AA with and without PNH clones or hypoplastic MDS.

After a comprehensive investigation, 105 patients were diagnosed with acquired AA (idiopathic AA), and 13 patients were diagnosed with AA and PNH clones, the median PNH clone sizes on granulocytes were 8.31% with a range (3.50%-87.04%), and the median PNH clone sizes on RBCs were 2.35% with range (0.12%-44.7%). Diagnosis of hypoplastic MDS (h-MDS) was confirmed in 8 patients.

The diagnosis and differentiation between h-MDS and AA were in accord to the criteria of the WHO classification of myeloid neoplasms, as well as recent recommendations and current standards[4, 5].

- The BM cellularity for diagnosis of our patients with hypoplastic MDS was BM cellularity **≤25%** which was determined in **7(87.5%)** patients and reduced age-**adjusted BM cellularity** which was determined in **one (12.5%)** patient.
- According to the WHO category, **6 (75%)** patients were diagnosed with MDS with multilineage dysplasia (**MDS-MLD**), and **2 (25%)** patients with MDS with single-lineage dysplasia (**MDS-SLD**).
- According to IPSS-R prognostic risk categories, **5(62.6%)** patients were diagnosed with **very low**, **3(37.5%)** patients were diagnosed with **low**, and one (**12.5%)** patient was diagnosed with **intermediate risk**.
- None of the 8 patients hMDS had positive PNH clones.

Selection of literature cohort.

To avoid duplication of cases we used the patient's unique ID or coding during selection and/ or selected the patients from the earliest publication. Also, we compared the patients' characteristics and ages between publications when the same author has many publications. Also, any publication that reported that the cases were published before was excluded.

We were unable to conduct a telomere length comparison between the literature cases due to the diverse methods used to measure the length and the different origins of the DNA (all leukocytes, granulocytes, lymphocytes, or buccal mucosa cells) utilized for this measurement.

Phenotypic Heterogeneity and Hematologic malignancy among BMF/AA/hMDS group.

This diverse cohort includes patients from 13 families. 20 patients (Table 1) presented with BMF/AA/MDS at age < 50 years with clinical symptoms suggestive of a IBMFS syndrome (i.e. microcephaly, short stature, skeletal or renal abnormalities, abnormal skin, nail, or hair). The median age was …. (range 14–55 years), and eight were male. 8 patients were positive for DEB, 7 developed hMDS with multilineage dysplasia (hMDS-MLD), 4 of them evolving to AML while the remaining one developed hMDS with single lineage dysplasia (hMDS-SLD). In additional their family members had strong history of MSD/AML, squamous cell carcinoma (SSC), and tumours of gastrointestinal tract, breast, genital or prostate cancer. WES and gene panel testing identifies a causative mutation in 3 of patients with DC (e.g. *TERT, DKC1*), while the remaining patient with DC feature has uninformative genetic panel testing resulting from unknown genetic cause. In this cohort, all patient developed hMDS-MLD with AML transformation occurrence in one patient. Also, they had strong family history of hepatocellular carcinoma (HCC), breast cancer or BMF/MDS/AML.

SUPPLEMENTAL METHODS

Laboratory investigations

A.Routine Hematological and Biochemical Investigations

A complete blood count was performed using Cell-Dyn 3700, the automated cell counter (Abbott diagnostic, Dallas, USA). Full complete blood count (CBC), calculation of RBCs indices, corrected reticulocyte count, and peripheral blood smear (PBS). Liver and renal function tests (LFTs), bilirubin total and direct bilirubin, blood sugar, and lipid profiles were performed on Cobas c 311& modular P auto analyzer (Roche Diagnostics, Mannheim, Germany). Testing viral hepatitis (HBV&HCV) and HIV was performed on the Architect platform (Abbott Laboratories, Chicago, Illinois, USA). Serum ferritin, iron study, serum lactate dehydrogenase (LDH), direct antiglobulin (Coomb's) test, serum lipids levels, CRP, LDH, and HbA1c were evaluated. A fully automated hemostasis benchtop analyzer (Sysmex Europe GmbH CA-1600, Germany) was used for a wide range of hemostasis tests. Prothrombin time, activated partial thromboplastin time, and D-dimer levels were among the tests performed. Serological tests for HIV, CMV, and EBV viruses were done to exclude viral infections. Serum immunoglobulins IgG, IgA, IgM, IgD, and IgE were measured by standard assays. Hemoglobin and serum protein electrophoresis, immunofixation, serum amyloid A protein, and β2 microglobulin were measured by standard assays.

Plasma concentrations of creatinine, calcium, phosphate, magnesium, 25-OH-vitamin D (S-25-OHD), and alkaline phosphatase (ALK.PH) were determined by standard assays. Plasma parathyroid hormone (PTH), thyroid stimulating hormone (TSH), cortisol level, and serum concentrations of follicle-stimulating hormone (FSH), luteinizing hormone (LH), and estradiol in (females) and testosterone in (males) were measured by standard assays.

B. Autoimmune disorders screening

All the patients were screened for connective tissue disease or other autoimmune disorders by creatine phosphokinase level, antinuclear antibodies (ANA), anti-double stranded DNA (anti-dsDNA), nucleosome antibodies, histones antibodies, Sm antibodies, RNP (68 kD/A/C) antibodies (Anti-U1/RNP), Sm/RNP antibodies, SS-A/Ro 60KD antibodies, SS-A/Ro52KD antibodies, SS-B/La antibodies, Scl70 antibodies, RNA-Polymerase III antibodies, Ku antibodies, PM-Scl 100 antibodies, Mi-2 antibodies, Jo-1 antibodies, CENP-A/B antibodies, PCNA antibodies, Ribosome P0 antibodies, DFS-70 antibodies, Lupus anticoagulant, Anticardiolipin (IgG or IgM), Anti–beta-2 glycoprotein I antibodies (IgG or IgM), complement proteins C3 and C4, rheumatoid factor (RF), anti-citrullinated Abs (ACPA), anti-neutrophil cytoplasmic Abs(Perinuclear-ANCA, Cytoplasmic-ANCA), Alb/creatinine ratio.

C. Flow cytometry analysis for detection of leukocyte subsets, paroxysmal nocturnal hemoglobinuria in leukocytes/RBCs/platelets and colonality of lymphocytes subsets

Analysis of bone marrow samples and peripharal blood using flow cytometry were performed to determine lymphocyte subsets as previously described [6]. T cells (CD3+) and the following T-cell subsets were examined CD3 T cells (Ts), CD8 cytotoxic T cells, CD4 helper T cells and CD3+CD56 NK cells. Gating using CD19 postive cells to determine Fc epsilon RII (FcεRII) expression (CD23) on B cells (Bs). Gating using CD16, CD56 for detection natural killer (NK) cells. Also, leukocyte subsets were examined to exclude monoclonality of lymphatic cells using CD2, CD3, CD5, CD4, CD7, CD8, CD10, CD11c, CD13, CD16, CD19, CD20, CD22, CD23, CD45, CD56, CD79b, CD123, HLA-DR, TCR-Alpha/Beta& gamma/delta, Kappa and Lambda and to exclude clonal evolution.

All patients were screened for presence of PNH. PNH clones were screening using heparinized PB from all patients stained with monoclonal antibodies (mAbs) specific for CD11b in granulocytes (Gs) and platelets. Glycophorin-A CD45(-) erythroid cells were analyzed using CD235a mAbs. FLAER and/or CD55 and CD 59 monoclonal antibodies were used to detect PNH clones between the previous mentioned cells. After staining, cells were washed in PBS and resuspended in FACS buffer and analyzed in a FACSCanto II instrument (Becton Dickinson, Franklin Lakes, NJ, USA) and obtained data were further analyzed with the FlowJo software package, version 10.0.7 (Treestar, Ashland, OR, USA).

Standard Cytogenetic Analysis

For cytogenetic analysis, bone marrow aspirates and blood were processed. The chromosomes were G-banded using trypsin and Wright stain. A total of twenty metaphase spreads were studied. The International System for Human Cytogenetic Nomenclature 2016 recommendations were followed for describing karyotypes[7].

Diepoxybutane chromosomal breakage

The method for the diepoxybutane (DEB) chromosomal breakage test has been previously described in detail [8]. In brief, whole blood (0.5 ml) was cultured in RPMI 1640 medium supplemented with 15% FCS, antibiotics, and 1% phytohemagglutinin (PHA) and incubated for 72 or 96 hours at 37°C in a 5% CO2 atmosphere with high humidity. For DEB experiments, duplicate cultures were generated, as well as a duplicated set of cultures to act as untreated controls. DEB DEB ((±)-1,2:3,4-diepoxybutane), at a final concentration in the medium of 0.1 μg/ml,, at a final concentration in the medium of 0. 1 g/mL, was added to the treated cultures 24 hours after their initiation, thus exposing cells to the chemical for 48 to 72 hours. Just before adding DEB to cell cultures, dilutions were made. After 72 to 96 hours, the cultures were harvested and chromosomal preparations were performed

according to standard procedures. For each culture, four microscope slides were prepared and stained with Giemsa. According to the International System for Human Cytogenetic Nomenclature, at least 50 metaphase cells per individual were evaluated and scored for chromosome number, as well as the number and type of chromosome and chromatid abnormalities (ISCN) [7].

DNA extraction

Blood samples and Buccal mucosa samples from the patients and control healthy voulnters were collected into 10 ml heparinized tubes DNA extraction. Genomic DNA was obtained of whole blood using a commercially available kit according to the manufacturer's instructions (QIAamp DNA extraction kit; Qiagen, Hilden, Germany). In some patients, peripheral blood nucleated cells and BM cells were also used for DNA extration.

Whole exome sequencing and data analysis

The family members DNA samples (extrated from blood and buccal mucosa) were sent to 3billion Laboratory (3billion Inc., Seoul, South Korea) or Kyoto University (Japan) for medical purposes to help diagnose the underlying genetic aetiology. Written informed consent for genetic investigation, and research was provided by the patients or their relatives in accordance with the Declaration of Helsinki.

A flagging mechanism showing a list of suspected and known IBMF genes was included as a helping tool to securely identify IBMF-causal mutations. Furthermore, the comprehensive WES data were manually examined for putative novel IBMF-causing genes. The pathogenic effect of the variations was determined using scoring systems and guidelines. Several tools were used to assess the deleteriousness of the variants (Supplemental Table S2-S6), such as Mendelian Clinically Applicable Pathogenicity (M-CAP) score, and rare exome variant ensemble learner (REVEL) score.(M-CAP: http://bejerano.stanford.edu/mcap/,REVEL:https://sites.google.com/site/revelgenomic). Eigen score was also used to have an integrated view of prediction scores (https://pubmed.ncbi.nlm.nih.gov/26727659/). Also using the Sherloc criteria, that refined the American College of Medical Genetics and Genomics (ACMG) guidelines to comprehensively assess variants' pathogenicity based on both clinical and functional evidence, attributing points to score each variant for pathogenicity. The point score thresholds for pathogenic and likely pathogenic variants are four (4P) and five points (5P), respectively[9].

Telomere length was measured by quantitative PCR (qPCR)

Telomere length was determined using using Absolute Mouse Telomere Length Quantification qPCR Assay Kit (Cosmo Bio Co., Lyd, Japan) for DNA samples taken at the time of diagnosis, as described previously [10]. Telomere length was determined in blood samples collected from all the family members before treatment with danazol. The Telomere lengths of patients were compared to distribution curves produced from best fit analysis of telomere lengths from healthy controls who were similarly measured by qPCR for age-matched analysis. The curve was adjusted for the 1st, 10th, 50th, 90th, and 99th percentiles. Telomere length that fell below the 1st or 10th percentile of age-matched controls were classified as extremely short or very short, respectively.

Radiological assessment:

A. Skeletal survey was routinely performed for bone disease and congenital abnormalities screening and skeletal/ dental abnormalities
B. Echocardiography was routinely performed for heart diseases and congenital abnormalities screening
C. Abdominal ultrasound and doppler: For optimal views of the liver, GB, kidneys, spleen, and pancreas. the portal vein, and other abdominal great vessels, ascites, and lymphadenopathy.
D. Computerized tomography (CT) brain and chest and pulmonary angiography as radiological assessment for brain, chest, malignancy, brain anomalies, cerebellar hypoplasia, and calcified falx cerebri, and interstitial lung disease.
E. Abdominal and pelvic angio-CT scans were performed to detect any abnormalities in abdominal and pelvic organs and liver pathology.
F. Magnetic resonance imaging (MRI) of the long bones and spine was performed for patients with fragility fractures.
G. Fundus examinations were performed to exclude any retinal abnormalities.
H. A whole-body positron emission tomography (PET) /CT was conducted to rule out the possibility of cancer, and the results were negative for active primary neoplastic illness (for example, PET/CT of the proband-1 showed extensive non-FDG avid lytic Osseous lesions including pelvic, femurs and right. humerus and tibia bones with multiple cortical interruption and fixation of both femurs suggestive of the benign process likely for further assessment, no malignancy.
I. Whole body Dual-phase bone scan with 99mTc-MDP was performed for 2 patients with acquired AA and vertebral lesions.

Dual-energy X-ray absorptiometry

Dual-energy X-ray absorptiometry (DXA) using the Lunar DPX DXA system (analysis version 14:10) pencil-beam scanner, manufactured by (GE Healthcare, USA) was used for bone mineral density (BMD). Sites measured were total body (TB), lumbar spine [bone mineral content (BMC [g]), BMD [aBMD (g/cm^3)], bone area [BA (cm^2)], proximal femurs, and forearms. The machine was calibrated daily according to the manufacturer's instructions, and service engineers regularly reviewed the calibrations. All scans and analyses were performed by the same operator. All scans and analyses were performed by the same operator. BMD were reported as a Z-score, which is the standard deviation above or below the age and gender matched mean reference value. Weight, height, age, and sex of each patient were used to estimate BMD and compared with the BMD values of controls as the previously published reference data for children and young adults [11-13].

Standing height using a portable stadiometer was measured to the nearest millimeter and weight was measured using an electronic scale to the nearest 100 grams. Body mass index (BMI) was computed by dividing weight in kilograms by height in meter squares. Subsequently, the height, weight and BMI were converted to Z scores using Egyptian/WHO normative data.

Histopathological studies (Hematoxylin and Eosin staining of BM biopsies)

Hematoxylin and Eosin (H&E) staining of bone marrow biopsies were prepared from the three affected siblings. The tissue sections were stained with Hematoxylin and Eosin (Hematoxylin, SR.No. AL2360, Batch No. HS502 & Eosin C.No. E30809,B.No.602023, Alpha chemika, India) as described previously. Also, immunohistochemistry was done using anti CD138, CD3, CD20, CD1a, kappa & lambda and S100 for bone biopsy obtained from patients with acquire AA and vertebral lesions.

Treatment of family 3 siblings with BMF

During the initial fractures (at a local hospital), many treatment modalities, such as vitamins supplement, platelets transfusion, steroids, and intravenous immunoglobulin (IVIG) were given to proband-1&II with partial improvement of platelets count. The pancytopenia of proband-1&II their sister (proband-III) firstly was treated by thrombopoietin receptor agonists (TPO-RA), and granulocyte colony-stimulating factor (GSF) for neutropenia. The patients' diseases were reclassified from acquired BMF to inherited BMF at Sohag University hospital. These treatments were discontinued after the WES results. The patients were given a variety of treatment options, including danazol, TPO-RA, and, Vit D3, and packed red-cell transfusion (RCT) or platelets depending on the severity of the anemia or thrombocytopenia. Based on dietary interview, all the patients had a sufficient intake of calcium (diet and supplements included). After 36 months of

zoledronic acid, none of the patients had motor disabilities and the level of physical activity was considered to be normal. Calcium and zoledronic acid treatments were started for the mothers who suffered from bone pain and progressive osteopenia 6 months ago.

Regarding the modalities of therapeutic intervention,

- Hematopoietic stem cell transplantation (HSCT) was planned for proband-III but eventually delayed because of the unavailability of non-carrier siblings or unrelated donors.

Follow up

Families were diligently followed during scheduled outpatient visits for the time of writing this manuscript. During this period, complications, radiological and laboratory profiles including blood counts and changes in serum Ca, P, PTH, Vit D3, Mg, IgE levels, LDH, amyloid A., and beta2 macroglobulin were checked. Follow-up ultrasonographic examinations and pulmonary function tests were performed according to indication. All patients were scanned by echocardiography and examined by ultrasound to view the liver, spleen, pancreas, and kidney size. The events of interest were recorded, such as frequency of infection, hospitalization, bleeding, the severity of pancytopenia or any abnormality of blood picture parameters, and progression of BMF, clonal evolution or haematological malignancy as well as the occurrence of new non-traumatic fractures, healing of the old fractures, bone mineral density changes, bone pain, lung, liver or renal affection, platelets, and RBCs transfusion frequency. They were instructed to report to our hospital if they had experienced new or unusual symptoms that could indicate bleeding, skin lesions, bone pain/fractures, and severe infection.

Treatment and follow up of patients with conventional AA

The patients were given a variety of treatment options, including danazol (oral, 200 mg twice per day), and packed red-cell transfusion (RCT) or platelets depending on the severity of the anemia or thrombocytopenia. Thrombopoietin receptor agonists (TPO-RA), and granulocyte colony-stimulating factor (GSF) for neutropenia were also used. Zoledronic acid IV infusion per 6 months, calcium, and vitamin D3 was used for patients with fragility fractures. Based on the dietary interview, all the patients had a sufficient intake of calcium (diet and supplements included). After 24 months of zoledronic acid, none of the patients had motor disabilities and the level of physical activity was normal without non-traumatic fracture events.

Hematopoietic stem cell transplantation and follow up of patients.

Twenty one patients were referred to the HSCT centers but 8 only had available matched siblings. Zoledronic acid IV infusion per 6 months, calcium, and Vit D3 was used for patients with vertebral lesions. Based on the dietary interview, all the patients had a sufficient intake of calcium (diet and supplements included). All patients were followed up for at least 6 months after AA diagnosis. After one year of zoledronic acid, none of the patients with vertebral lesions had motor disabilities and the level of physical activity was normal without non-traumatic fracture events.

Statistical Analysis

Statistical analysis was performed by SPSS 18.0 software (SPSS, Inc., Chicago, IL) and GraphPad Prism. Quantitative data were represented as mean ± standard deviation (SD) or median (interquartile (25th, 75th percentile) range), and as relevant for informative purposes, range (minimum to maximum), and the Pearson $\chi2$ test or Fisher exact test were used to compare differences of dichotomous variables. The variables were compared using non-parametric tests, a Student unpaired t-test, one-way ANOVA, or a $\chi2$ test. Comparisons between or among groups were carried out by t-test or analysis of covariance, respectively, with post hoc pairwise comparisons corrected with the Bonferroni method. Statistical significance was assumed at the P-value was < 0.05 in all analyses. The figures were performed using the GraphPad Prism software package, version 5.02 (San Diego, CA).

Table (S1) Summary of demographic, clinical, and molecular observations in individuals with Cernunnos deficiency

Pt	Sex/AOD (Months)	Zygosity/variant type/ affected exon(s) or intron(s)	Clinical/anomalies/ Malignancy	Treatment/ outcomes	References
1	F/72	Homozygous/Frameshift dup/ Exon 2, c.11dupT (p.E5Gfs*43)	Immunodeficiency	BMT/Alive	[14] (Dai, et al. 2003)
2	-/168	Compound heterozygous/ Missenses; missense/Exon 2; exon3, c.169C>G (p.R57G);c.367T>C (p.C123R)	Microcephaly, Mental/ Urogenital and bone malformation/Facial dysmorphia, Retardation/AIHA./ ITP	IS, IGRT, Splenectomy/ Deceased(18y)	[15] (Buck, et al. 2006)
3	-/ 24	Homozygous/Nonsense/ Exon 5c.532C>T (p.R178*)	Microcephaly/ Recurrent RTI and GI tract infections	IGRT / Deceased (4)y	[15] (Buck, et al. 2006)
4	M/156	Homozygous/Indel/Intron 2 c.177+1_177+ 3delGTAinsTT	Microcephaly/ Facial dysmorphia, Bone Malformation/ AIHA. ITP	IGRT /Alive	[15] (Buck, et al. 2006)
5	F/24	Homozygous/Indel/Intron 2 c.177+1_177+ 3delGTAinsTT	Microcephaly/ Facial dysmorphic, Bone malformation	IGRT Alive	[15] (Buck, et al. 2006)
6	M/84	Homozygous/Missense/ Exon 2 c.169C>G (p.R57G)	Microcephaly / Facial dysmorphia /BMF	IGRT / Alive	[15] [16] (Faraci, et al. 2009) (Buck, et al. 2006)
7	M/144	Homozygous/Missense/ Exon 2 c.169C>G (p.R57G)	Microcephaly/ Facial dysmorphia	-/Alive	[17](Dutrannoy, et al. 2010)
8	M/72	Compound heterozygous/ Frame shift dup; large deletion/Exon 4; exons 2 and 3 c.495dupA (p.D166Rfs* 20); 1.9 kb deletion	Microcephaly/ Hepatosplenomegaly, Abdominal Lymphadenopathy	IGRT /Alive	[17, 18] (Dutrannoy, et al. 2010), (Meyer-Bahlburg, et al. 2014)
9	M/24	Compound heterozygous/ Fame shift dup; large deletion/Exon 4; exons 2 and 3 c.495dupA (p.D166Rfs* 20); 1.9 kb deletion	Recurrent RTI	-/Alive	[17](Dutrannoy, et al. 2010)
10	M/96	Compound heterozygous/ Nonsense; large deletion/Exon 4; exons 1–3 c.526C>T (p. R176*); 6.9 kb deletion	Microcephaly/ Facial dysmorphia, Clinodactyly/ITP	IGRT/ Alive	[17](Dutrannoy, et al. 2010)
11	M/12	Homozygous/Nonsense/ Exon 5, c.532C>T (p.R178*)	Microcephaly/ dystrophy and mouth lesions/AIHA	Blood transfusions, BMT /Deceased (1.5y)	[17](Dutrannoy, et al. 2010)
12	F/20	Homozygous/Nonsense/ Exon 5, c.532C>T (p.R178*)	Microcephaly/ Recurrent RTI, Mucocutaneous candidiasis,	IGRT, AB prophylaxis Deceased (4.5y)	[19] (Turul, et al. 2011)
13	-/72	Homozygous/Nonsense/ Exon 4 c.501C>A (p.Y167*)	Microcephaly/ Recurrent RTI	-/ Alive	[20] (Du, et al. 2012)

14	M/24	Homozygous/Nonsense/ Exon 2, c.169C>T (p.R57*)	Microcephaly/ Episodic diarrhea	-/ Alive	[20] (Du, et al. 2012)
15	M/132	Homozygous/Large deletion/Exons 2–5 ex 2–5 deletion	Microcephaly/ Microphthalmia, Blepharophimosis, posterior cleft palate, acral anomalies	IGRT/ Alive	[20, 21] (Du, et al. 2012) (Verloes, et al. 2001)
16	F/1	Homozygous/Nonsense/ Exon 5 c.532C>T (p.R178*)	Microcephaly/ Facial dysmorphia/ Diarrhea	BMT/ Alive	[22] (Çağdaş, et al. 2012)
17	F/9	Homozygous/Nonsense/ Exon 5, c.532C>T (p.R178*)	Microcephaly/ Facial dysmorphia/ CMV pneumonia	IGRT, IS, AB prophylaxis, BMT Alive	[22] (Çağdaş, et al. 2012)
18	F/36	Homozygous/Nonsense/ Exon 5, c.532C>T (p.R178*)	Microcephaly/Facial dysmorphia, Polydactyly/BMF	IGRT, BMT (candidate)/ Alive	[23] (Cipe, et al. 2014)
19	M/7	Homozygous/Nonsense/ Exon 5, c.532C>T (p.R178*)	Microcephaly/Facial dysmorphia /BMF/ **NHL**	Anti-epileptic, Chemotherapy (RTX &MTX), IGRT/Alive	[24] (Patiroglu, et al. 2015)
20	F/42	Homozygous/Nonsense/ Exon 5, c.532C>T (p.R178*)	Microcephaly/ Facial dysmorphia/Pancytopenia	IGRT, AB prophylaxis /Alive	[25] (Akar, et al. 2016)
21	-/48	Homozygous/Nonsense/ Exon 4, c.501C>A (p.Y167*)	Microcephaly/ Recurrent RTI	BMT/ Alive	[26] (H, et al. 2016)
22	-/1.5	Homozygous/Nonsense/ Exon 5, c.532C>T (p.R178*)	Microcephaly/ Diarrhea	BMT /Alive	[26] (H, et al. 2016)
23	-/15	Homozygous/Nonsense/ Exon 2, c.169C>T (p.R57*)	Microcephaly/ UTI, RTI	BMT/ Alive	[26] (H, et al. 2016)
24	-/120	Homozygous/Nonsense/ Exon 5, c.532C>T (p.R178*)	Microcephaly/ RTI	BMT /Alive	[26] (H, et al. 2016)
25	-/96	Homozygous/Frameshift dup/Exon 3, c.324dupG (p.R109Afs*3)	Microcephaly	BMT(candidate) /Alive	[26] (H, et al. 2016)
26	-/11	Homozygous/Nonsense/ Exon 5, c.532C>T (p.R178*)	Microcephaly/ Recurrent fungal RTI	BMT(candidate) /Alive	[26] (H, et al. 2016)
27	-/96	Homozygous/Nonsense/ Exon 5, c.532C>T (p.R178*)	Microcephaly/ Recurrent RTI	BMT(candidate) /Alive	[26] (H, et al. 2016)
28	-/108	Homozygous/Nonsense/ Exon 5, c.532C>T (p.R178*)	Microcephaly/ RTI, Diarrhea	BMT /Alive	[26] (H, et al. 2016)

29	-/12	Homozygous/Nonsense/ Exon 5, c.532C>T (p.R178*)	Microcephaly / BCGitis, Otitis/ Autoimmunity	BMT/ Alive	[26] (H, et al. 2016)
30	M/48	Homozygous/Nonsense/ Exon 2, c.169C>T (p.R57*)	RTI/ Bone marrow aplasia, Pancytopenia Short telomere	IGRT/BMT Deceased (8y, Severe complications after HSCT)	[27] (Carrillo, et al. 2017)
31	M/180	Homozygous/Canonical splice site/Intron 3, c.390+1G>C	Microcephaly/Facial dysmorphia Radial deviation of little fingers/ Pancytopenia	IGRT, AB prophylaxis Alive	[28] (Sheikh, et al. 2017)
32	F/2	Homozygous/Canonical splice site/Intron 3, c.390+1G>C	Microcephaly, muscle spasms, mouth pulling to one side, progressive ataxia, worsening proximal muscle weakness, and inability to walk without assistance/Facial dysmorphia/ Pancytopenia	IGRT, AB Prophylaxis/ Deceased (20 y, Septic shock)	[28] (Sheikh, et al. 2017)
33	M/12	Homozygous/Canonical splice site/Intron 3. c.390+1G>C	Microcephaly, Neurological manifestation/Facial dysmorphia Pancytopenia	No IGRT due to Anaphylaxis/ Alive	[28] (Sheikh, et al. 2017)
34	F/36	Homozygous/Nonsense/ Exon 5, c.526C>T (p.R176*)	Microcephaly/ AIHA/ ITP/ Neonatal hypothyroidism, Splenomegaly, Lymphadenopathy	Levothyroxine, IGRT BMT / Alive	[29] (Yazdani, et al. 2017)
35	M/256	Homozygous/Missense/ Exon 3 c.236T>C (p.L79P)	Microcephaly/Skeletal anomalies (clinodactyly)/ **MDS (Initially monosomy 7, replaced by del (20))**	-/Alive	[30] (Kager, et al. 2018)
36	F/4.5	Homozygous/Nonsense/ Exon 2 c.169C>T (p.R57*)	Microcephaly	BMT/ Alive	[31] (Recio, et al. 2018)
37	F/94	Homozygous/Nonsense/ Exon 2, c.169C>T (p.R57*)	Microcephaly/ Facial dysmorphia/ITP	BMT/ Alive	[31] (Recio, et al. 2018)
38	F/30	Homozygous/Nonsense/ Exon 5, c.532C>T (p.R178*)	Microcephaly/AIHA	IS / Alive	[32] (Esmaeilzadeh, et al. 2019)
39	F/18	Homozygous/Nonsense/ Exon 5, c.532C>T (p.R178*)	-	BMT / Alive	[33] (Firtina, et al. 2020)
40	F/66	Homozygous/Nonsense/ Exon 5, c.532C>T (p.R178*)	-	BMT/ Alive	[33] (Firtina, et al. 2020)
41	M/3	Homozygous/Missense/ Exon 3, c.367T>C (p.C123R)	Microcephaly/ Facial dysmorphia, Ectopic kidney/AIHA/ G6PD def.	Blood transfusion, IS, adenosine and cardioversion, IGRT/Deceased	[34] (Al-Marhoobi, et al. 2020)

42	M/204	Homozygous/Canonical splice site/Intron 4, c.530-2A>T	**AML**	-	[35] (Arunachalam, et al. 2021)
43	-/108	Homozygous/Missense/ Exon 2, c.169C>G (p.R57G)	Facial dysmorphia/BMF	BMT / Alive	[36] (Miano, et al. 2021)
44	-/144	Homozygous/Missense/ Exon 4, c.506A>T (p.E169V)	Facial dysmorphia/BMF	BMT / Alive	[36] (Miano, et al. 2021)
45	-/180	Homozygous/Missense/ Exon 4, c.506A>T (p.E169V)	Facial dysmorphia/BMF	BMT / Alive	[36] (Miano, et al. 2021)
46	M/10	Homozygous/Frameshift/ del/Exon 5 c.544_545delGA (p.E182Tfs*3)	Recurrent gastroenteritis, AIHA Pneumonia, CMV infection	IS	[37]
47	M/11	Homozygous/Frameshift/ del/Exon 3, c.221_222delGT(p.C74Sfs*4)	Skin pustule and abscess, Oral Thrush/ Generalized erythematous macular rash	-	[37] (Vignesh, et al. 2020)
48	F/2	Homozygous/Canonical splice site/Intron 3, c.390+1G>T	Microcephaly, dd, bilateral mild Sensorineural hearing loss/ Oral thrush, BCGitis, Bilateral otitis/ Eczema, thymus atrophy, hypothyroidism	Levothyroxine, IGRT, BMT(candidate) /Alive	[38] (Farsi, et al. 2021)
49	F/6	Homozygous/Canonical splice site/Intron 3 c.390+1G>T	Microcephaly/ Oral candidiasis, Diarrhea, UTI, BCGitis, mastoiditis/ Bicytopenia	IGRT/ BMT (candidate) Deceased (11months, Septic shock)	[39] (Jamee, et al. 2022)
50	M/4	Homozygous frame shift c.A580Ins.T; p.M194fs	Microcephaly, triangle shaped face recurrent pneumonia, and failure to thrive clubbing	HSCT/Alive	[40] (Frizinsky, et al. 2022)

Abbreviations: AB; antibiotic, AIHA; autoimmune hemolytic anemia, AOD; age of diagnosis, BCG; bacillus Calmette–Guerin, BMF; bone marrow failure, BMT; bone marrow transplant, CMV; cytomegalovirus, dd; developmental delay, FH; family history, FTT; failure to thrive, GI; gastrointestinal, HTN ;hypertension, IEI; inborn error of immunity, IGRT; immunoglobulin replacement therapy, IS; immunosuppressive, ITP; immune thrombocytopenic purpura, IVIg; intravenous immunoglobulin, MDS; Myelodysplastic syndrome, MTX; methotrexate, NHL; non Hodgkin lymphoma, RTI; respiratory tract infection, RTX; rituximab, UTI; urinary tract infection.

* Siblings.

Table (S2) Summary of demographic, and type of malignancy in 216 individuals with Nijmegen Breakage Syndrome

	Number of patients	Sex	Complications/ Type of Malignancy	References
1	149 patients	72 Females<br>77 Males.	Eighty malignancies were diagnosed in 63* (42 %)<br>B-cell non-Hodgkin's lymphoma (B-NHL; n = 30),<br>T-cell non-Hodgkin's lymphoma (T-NHL; n = 21),<br>Hodgkin's lymphoma (cHL; n = 7),<br>T-cell acute leukemia (T-ALL; n = 10)<br>Undefined lymphoma/leukemia (n = 7).<br>Medulloblastoma (n = 2),<br>Neuroblastoma (n = 1),<br>Dysgerminoma (n = 1)<br>Thyroid cancer (n = 1).	[41]
2	One patient	Girl	Hodgkin lymphomas and Combined variable immunodeficiency	[42]
3	Two half sibs	2 Males	Peripheral T-cell lymphoma (n = 1)<br>Cortical T-cell acute lymphoid leukemia(n = 1)	[43]
4	56 patients	32 Males<br>24 Females	Lymphoma, the majority B cell. (n = 17)<br>Precursor T cell lymphoblastic lymphoma/leukemia (n = 2)<br>Leukemia (n = 1)<br>Medulloblastoma (n = 1)<br>Rhabdomyosarcoma (n = 1)<br>Glioma (n = 1)	[44]
5	One patient	Girl	Cutaneous tuberculosis	[45]
6	One patient	Boy	Recurrent respiratory infections	[46]
7	One patient	Girl	Febrile enteritis and a stomatitis followed by candidiasis	[47]
8	One patient	Girl	Chronic refractory necrotizing granulomatous ulcerations	[48]
9	Two patients	Boy<br>Girl	Developmental delay, and microcephaly without recurring infections<br>Short stature, microcephaly, hepatosplenomegaly, rectovaginal fistula, anal atresia, an ectopic kidney, and otitis media	[49]
10	Two patients	2 Males	T cell lymphoblastic lymphoma/leukemia (n = 2)	[50]

*Many patients have more than one malignancy (two to three types)

Table (S3) Summary of demographic, and type of malignancy in 46 individuals with DNA Ligase IV deficiency

	Age at dx (years)	Sex	Complications/ Type of Malignancy	References
1	4.5	Male	**Acute T-cell leukemia**	[51]
2	14	Female	**Epstein-Barr virus-associated B-cell lymphoma**	[52]
3	2	Female	Autoimmune hemolytic anemia and immune thrombocytopenia lymphoproliferation, opportunistic infection, interstitial nephritis	[53]
4	14	Male	**Acute lymphoblastic leukemia**	[54, 55]
5	26	Female	Seckel syndrome. Cough, fever,	[56]
6	9	Male	Dysmorphism, developmental delay and plantar warts	[57]
7	46	Male	Chronic skin conditions, photosensitivity, telangiectasia, sinusitis	[57]
8	48	Female	Dysmorphism, growth failure, respiratory infections, psoriasis	[57]
9	9	Female	Dysmorphism, dwarfism, mental delay, psoriasis	[57]
10	4.75	Male	Dysmorphism, developmental delay, hypogonadism, **leukemia**	[58]
11	1.5	Female	Respiratory infections, candidiasis, chronic diarrhea, failure to thrive	[59]
12	0	Female	Microcephaly/Alive after HSCT	[60]
13	2	Female	Chronic diarrhea, , failure to thrive, autoimmune cytopenia	[60]
14	1.5	Female	Pneumonia and sepsis/Died of EBV PTLD 50 days after HSCT	[61]
15	0	Female	Died of VOD 2 months after HSCT	[61]
16	1.3	Male	Pancytopenia, growth retardation, microcephaly, MDS	[62]
17	0.1	Female	Hepatosplenomegaly, lymphadenopathy, Alive after HSCT	[63]
18	10	Female	Sinopulmonary recurrent infections Alive after 2 RIC HSCTs	[56]
19	6	Male	Low weight, upper respiratory and urinary infections/ Alive	[56]
20	0.25	Male	Tubulopathy/ Died at 6 months of sepsis, respiratory insufficiency and severe gastrointestinal bleeding	[64]
21	34	Female	Rectorrhagia / Died of **metastatic anal cancer**	[64]
22	17.5	Female	Small cerebral aneurysm, primary ovarian failure/ Alive	[64]
23	11.75	Female	Atrial-ventricular septal defect, atrophic kidney, rib hypoplasia, fusion of carpal bones, primary ovarian failure./ Alive	[64]
24	7.8	Female	Anal atresia with rectovaginal fistula, esotropia.	[64]
25	2.1	Female	Unilateral congenital hip dysplasia, cutis marmorata./ Alive. BMT	[64]
26	2.5	Female	Psoriasis/ Alive	[64]
27	2	Male	Unilateral congenital hip dysplasia. / Alive	[64]
28	2	Female	Congenital hip dysplasia, 2/3 toe syndactyly, vomiting/ Alive	[64]
29	3.67	Female	Microcephaly, growth failure/ Alive	[64]
30	1.75	Female	Microcephaly, growth failure/ Alive	[64]
31	5.5		Microcephaly, hypopigmentation Hypopigmentation, hypermobile knees, single palmar crease, 2/3 toe syndactyly, sandal gap/ Alive	[64]
32	13	Female	Recurrent pneumonias and sinusitis<br>Recurrent respiratory infections, microcephaly, short stature./Alive	[65]
33	-	Male	Pneumonia /Alive	[65]
34	-	Female	Asymptomatic /Alive	[65]

35	2	Female	Ulcerated stomatitis, encephalitis, lung and brain lymphoma./Died	[66]
36	7	Female	Respiratory infections, pancytopenia Alive and well after HSCT	[67]
37	9	Male	Chronic diarrhea, hemorrhagic syndrome/ Died of aplasia at age 10	[67]
38	7	Female	Recurrent sinopulmonary infections, urosepsis, urofacial syndrome Died of pneumonia	[68]
39	6	Male	Recurrent upper respiratory infections, dysgammaglobulinemia/ Alive	[69]
40	0.75	Female	Pneumonia at 4 months Died of **lymphoma**	[70]
41	1.5	Male	Pneumonia at 12 months Died of **lymphoma**	[70]
42	1	Male	Avascular necrosis of femoral head, overweight. Alive and well	[70]
43	10	Female	Microcephaly, dysmorphism, developmental delay, recurrent infections, jaundice/ Died	[70]
44	10	Female	Microcephaly, dysmorphism, developmental delay, recurrent infections, jaundice/ Died	[70]
45	10	Male	Microcephaly, facial dysmorphism, **MDS and monosomy 7**	[71]
46	23	Male	Epstein-Barr virus-independent **diffuse large B-cell lymphoma**	[72]

•

Induction by 1,2,3,4-diepoxybutane (DEB)

P7 [Family -2] **P20 [Family -12]**

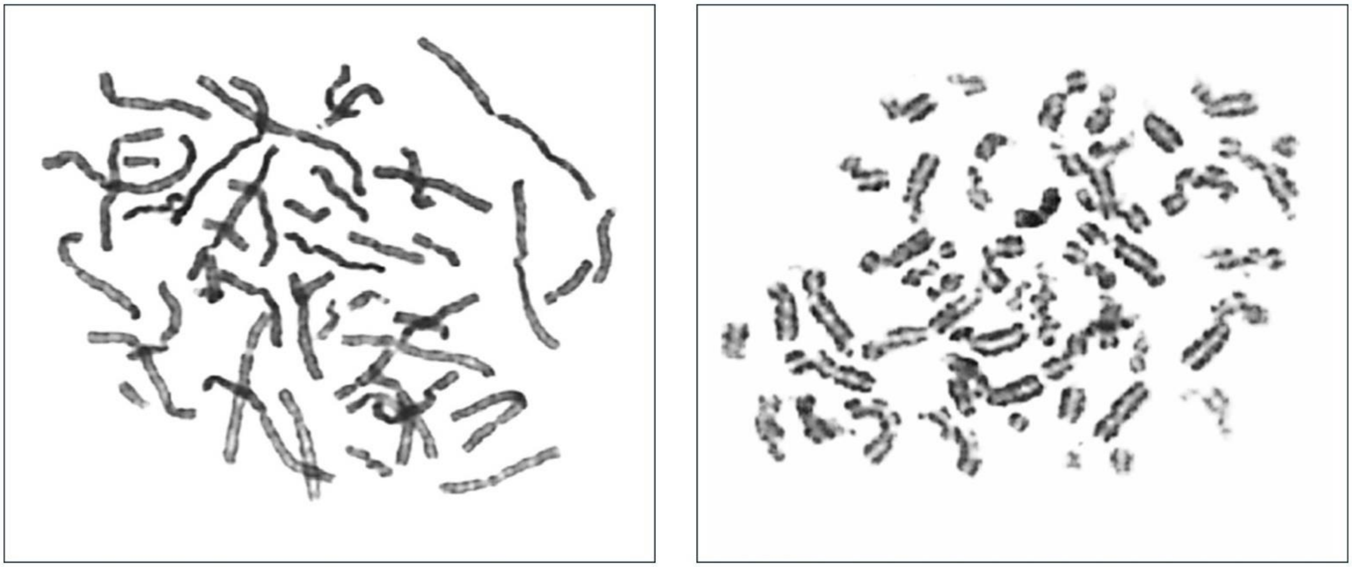

46-XY [Negative for breakage test]

Staining: Giemsa
Specimen type: Peripheral blood
Number of cultures:4
Metaphases analyzed: 85

46-XX [Positive for breakage test]

Staining: Giemsa
Specimen type: Peripheral blood
Number of cultures:4
Metaphases analyzed: 160

Figure S2. Diepoxybutane (DEB) induced chromosomal breakage assay.

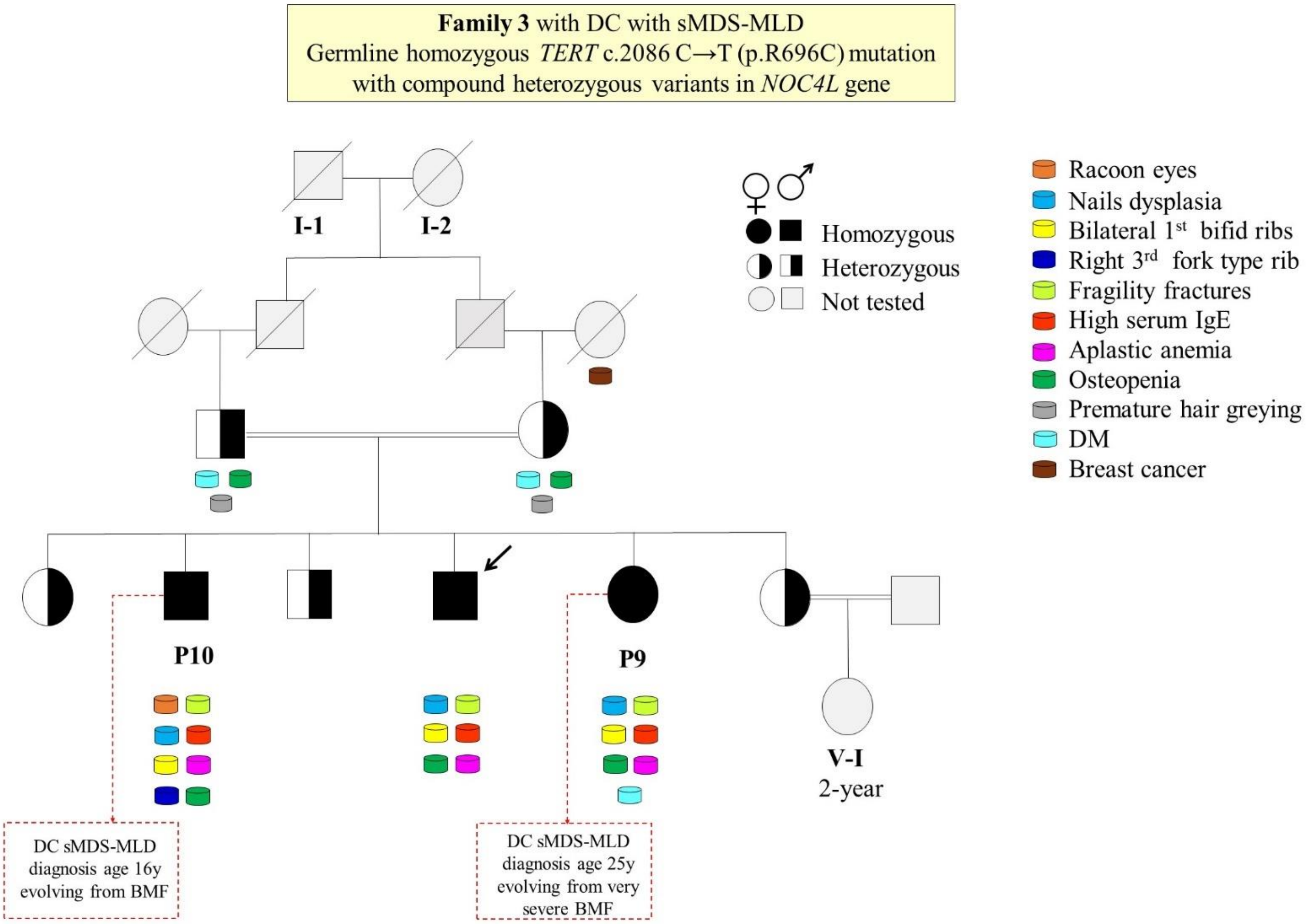

Figure S3. Pedigree, clinical features, malignancy type and molecular findings of family 3with *TERT* mutation in homozygous and heterozygous individuals.

Case III-B1	Case III-B5	Case III-B2

Hight

Stature-for-Age Percentiles: Egyptian Boys, 2 to 21 Years

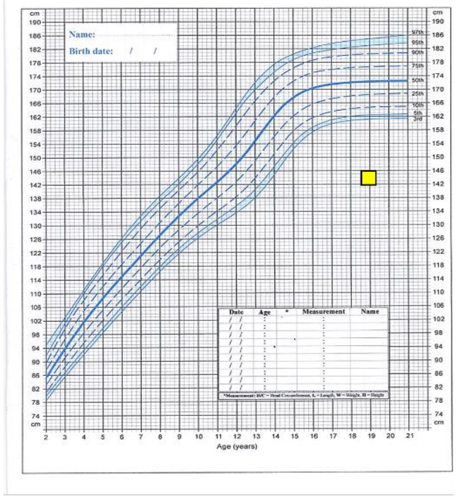

Stature-for-Age Percentiles: Egyptian Boys, 2 to 21 Years

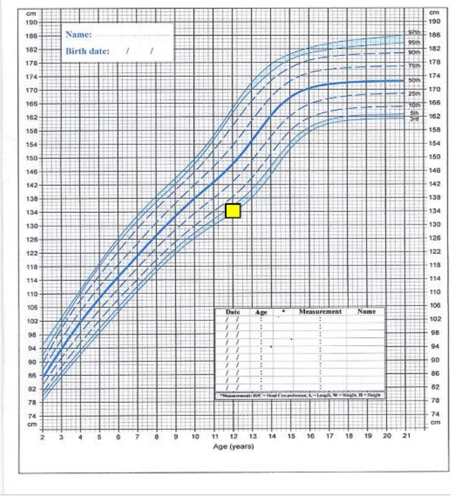

Stature-for-Age Percentiles: Egyptian Girls, 2 to 21 Years

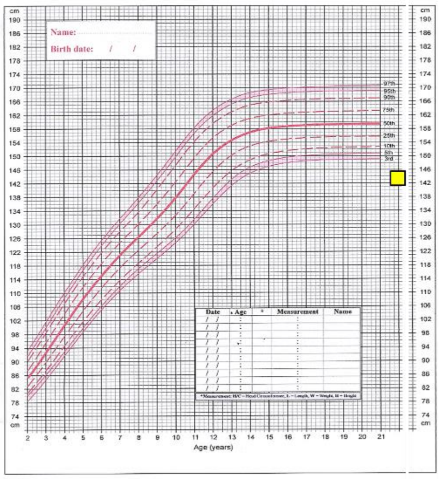

Weight

Weight-for-Age Percentiles: Egyptian Boys, 2 to 21 Years

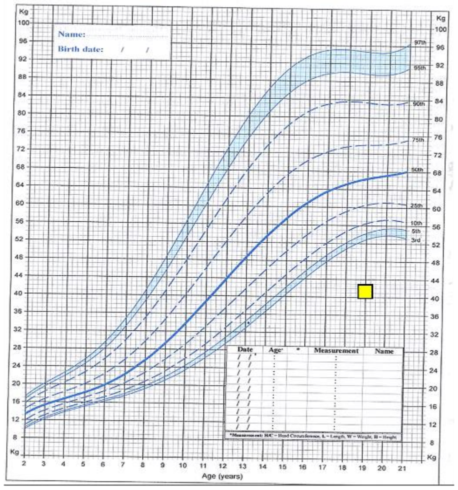

Weight-for-Age Percentiles: Egyptian Boys, 2 to 21 Years

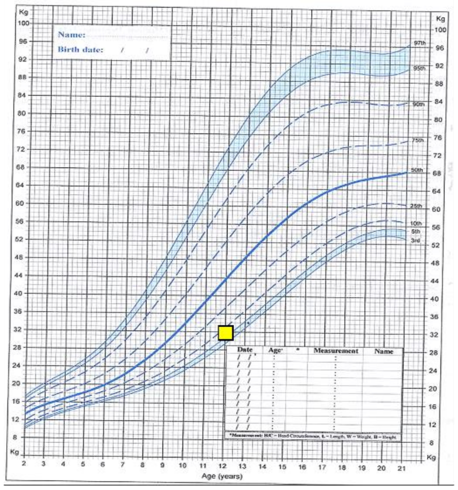

Weight-for-Age Percentiles: Egyptian Girls, 2 to 21 Years

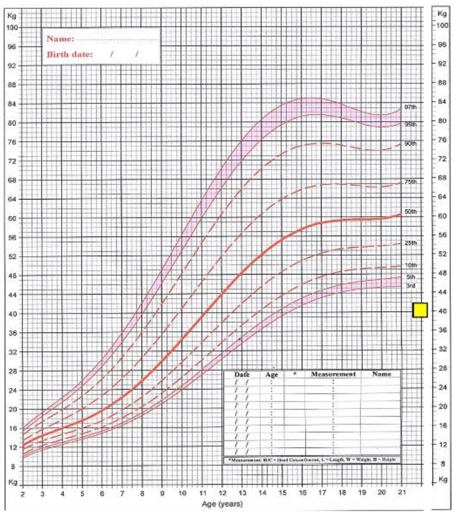

BMI

Body Mass Index-for-Age Percentiles: Egyptian Boys, 2 to 21 Years

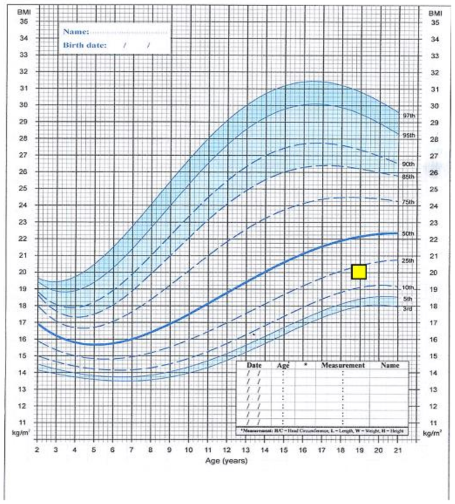

Body Mass Index-for-Age Percentiles: Egyptian Boys, 2 to 21 Years

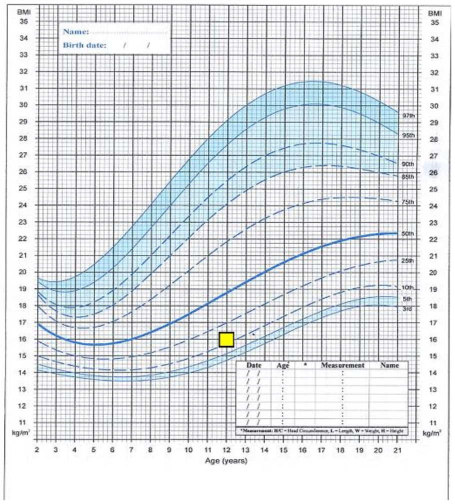

Body Mass Index-for-Age Percentiles: Egyptian Girls, 2 to 21 Years

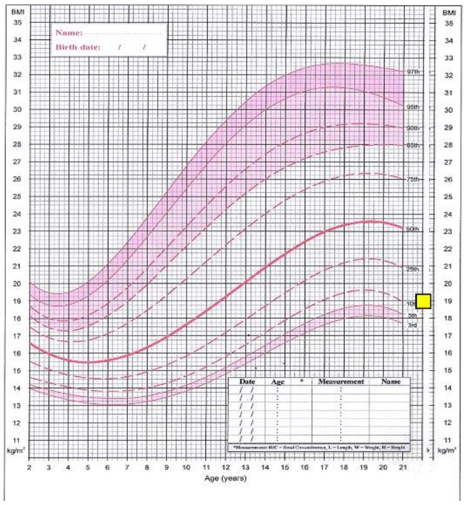

Case III-B1 **Case III-B5** **Case III-B2**

Head circumference

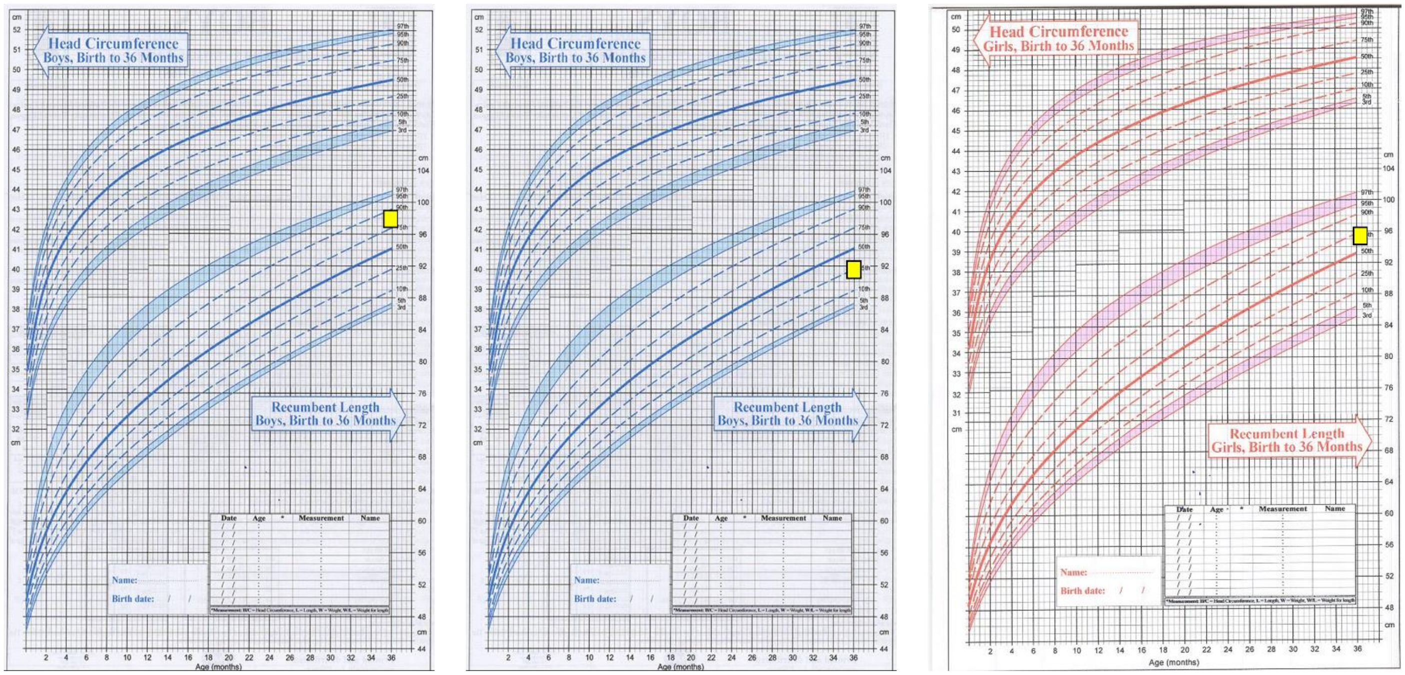

Figure S4. Growth curves of family's-1 cases. Yellow squares represent scores at age during the study. Hight and weight, head circumference all below or at the third percentile (3rd centile), BMI m (between the 5th and 25th centiles).

BMI; body mass index

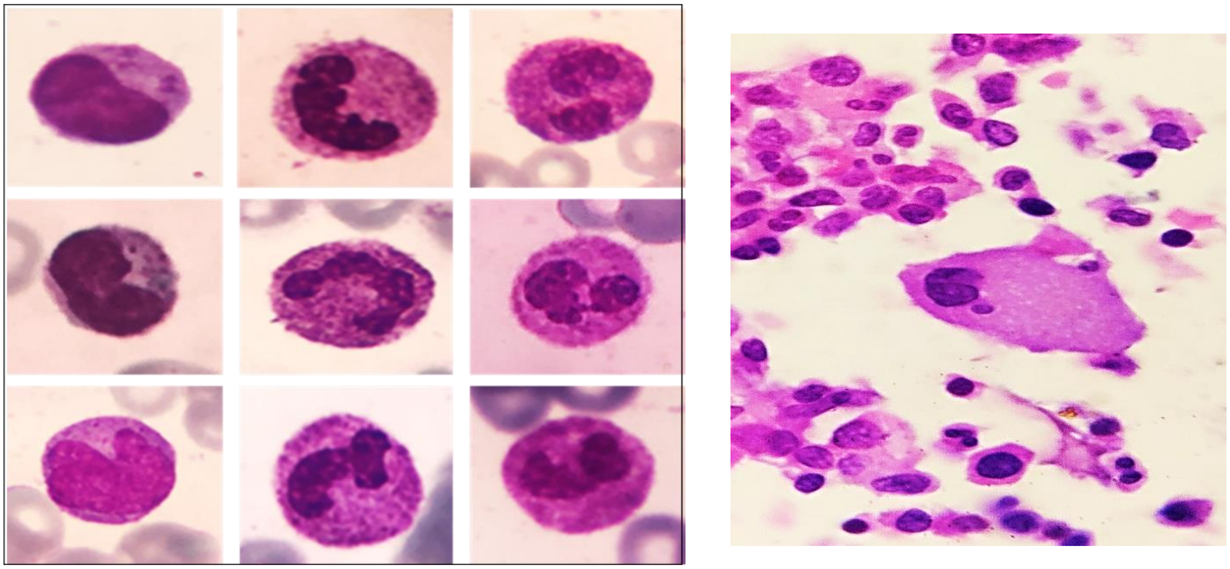

Figure S5, Progression of dysplasia in patient 2 with *NHEJI* deficiency

REFERENCES

[1] C. Sugimori, T. Chuhjo, X. Feng, H. Yamazaki, A. Takami, M. Teramura, H. Mizoguchi, M. Omine, S. Nakao, Minor population of CD55-CD59- blood cells predicts response to immunosuppressive therapy and prognosis in patients with aplastic anemia, Blood, 107 (2006) 1308-1314.
[2] B.M. Camitta, What is the definition of cure for aplastic anemia?, Acta haematologica, 103 (2000) 16-18.
[3] J.C. Marsh, S.E. Ball, J. Cavenagh, P. Darbyshire, I. Dokal, E.C. Gordon-Smith, J. Keidan, A. Laurie, A. Martin, J. Mercieca, S.B. Killick, R. Stewart, J.A. Yin, Guidelines for the diagnosis and management of aplastic anaemia, British journal of haematology, 147 (2009) 43-70.
[4] D. Arber, A. Orazi, R. Hasserjian, R. Brunning, M. Le Beau, A. Porwit, A. Tefferi, R. Levine, C. Bloomfield, M. Cazzola, Introduction and overview of the classification of myeloid neoplasms, WHO Classification of Tumours. Lyon, France: IARC, (2017) 16-27.
[5] J.M. Bennett, A. Orazi, Diagnostic criteria to distinguish hypocellular acute myeloid leukemia from hypocellular myelodysplastic syndromes and aplastic anemia: recommendations for a standardized approach, haematologica, 94 (2009) 264.
[6] Y. Ding, L. Zhou, Y. Xia, W. Wang, Y. Wang, L. Li, Z. Qi, L. Zhong, J. Sun, W. Tang, Reference values for peripheral blood lymphocyte subsets of healthy children in China, Journal of Allergy and Clinical Immunology, 142 (2018) 970-973. e978.
[7] J. McGowan-Jordan, ISCN 2016: an international system for human cytogenomic nomenclature (2016); recommendations of the international standing human committee on human cytogenomic nomenclature including new sequence-based cytogenomic, Karger, 2016.
[8] A.D. Auerbach, A. Rogatko, T.M. Schroeder-Kurth, International Fanconi Anemia Registry: relation of clinical symptoms to diepoxybutane sensitivity, (1989).
[9] K. Nykamp, M. Anderson, M. Powers, J. Garcia, B. Herrera, Y.Y. Ho, Y. Kobayashi, N. Patil, J. Thusberg, M. Westbrook, S. Topper, Sherloc: a comprehensive refinement of the ACMG-AMP variant classification criteria, Genetics in medicine : official journal of the American College of Medical Genetics, 19 (2017) 1105-1117.
[10] J. Lin, D.L. Smith, K. Esteves, S. Drury, Telomere length measurement by qPCR–Summary of critical factors and recommendations for assay design, Psychoneuroendocrinology, 99 (2019) 271-278.
[11] S.A. Ibrahim, M.A. Samy, M.K. Matter, A.O. Saleh, Bone mineral density in Egyptian adolescents and adults with short stature: results of a national survey, Eastern Mediterranean health journal = La revue de sante de la Mediterranee orientale = al-Majallah al-sihhiyah li-sharq al-mutawassit, 17 (2011) 687-693.
[12] W. Högler, J. Briody, H. Woodhead, A. Chan, C. Cowell, Importance of lean mass in the interpretation of total body densitometry in children and adolescents, The Journal of pediatrics, 143 (2003) 81-88.
[13] N.Z. Hamoda, H.M. Eltokhy, E.F. Mohamed, D.S. Mohammed, Bone mineral density in young adult Egyptian women and its relations to different anthropometric measures, The Scientific Journal of Al-Azhar Medical Faculty, Girls, 4 (2020) 394.
[14] Y. Dai, B. Kysela, L.A. Hanakahi, K. Manolis, E. Riballo, M. Stumm, T.O. Harville, S.C. West, M.A. Oettinger, P.A. Jeggo, Nonhomologous end joining and V(D)J recombination require an additional factor, Proceedings of the National Academy of Sciences of the United States of America, 100 (2003) 2462-2467.
[15] D. Buck, L. Malivert, R. de Chasseval, A. Barraud, M.C. Fondanèche, O. Sanal, A. Plebani, J.L. Stéphan, M. Hufnagel, F. le Deist, A. Fischer, A. Durandy, J.P. de Villartay, P. Revy, Cernunnos, a novel nonhomologous end-joining factor, is mutated in human immunodeficiency with microcephaly, Cell, 124 (2006) 287-299.
[16] M. Faraci, E. Lanino, C. Micalizzi, G. Morreale, D. Di Martino, L. Banov, P. Comoli, F. Locatelli, A. Soresina, A. Plebani, Unrelated hematopoietic stem cell transplantation for Cernunnos-XLF deficiency, Pediatric transplantation, 13 (2009) 785-789.

[17] V. Dutrannoy, I. Demuth, U. Baumann, D. Schindler, K. Konrat, H. Neitzel, G. Gillessen-Kaesbach, J. Radszewski, S. Rothe, M.T. Schellenberger, G. Nürnberg, P. Nürnberg, K.W. Teik, R. Nallusamy, A. Reis, K. Sperling, M. Digweed, R. Varon, Clinical variability and novel mutations in the NHEJ1 gene in patients with a Nijmegen breakage syndrome-like phenotype, Human mutation, 31 (2010) 1059-1068.
[18] A. Meyer-Bahlburg, F. Dressler, U. Baumann, Chronic arthritis in a boy with Cernunnos immunodeficiency, Clinical immunology (Orlando, Fla.), 154 (2014) 47-48.
[19] T. Turul, I. Tezcan, O. Sanal, Cernunnos deficiency: a case report, Journal of investigational allergology & clinical immunology, 21 (2011) 313-316.
[20] L. Du, R. Peng, A. Björkman, N. Filipe de Miranda, C. Rosner, A. Kotnis, M. Berglund, C. Liu, R. Rosenquist, G. Enblad, C. Sundström, M. Hojjat-Farsangi, H. Rabbani, M.R. Teixeira, P. Revy, A. Durandy, Y. Zeng, A.R. Gennery, J.P. de Villartay, Q. Pan-Hammarström, Cernunnos influences human immunoglobulin class switch recombination and may be associated with B cell lymphomagenesis, The Journal of experimental medicine, 209 (2012) 291-305.
[21] A. Verloes, M.F. Dresse, H. Keutgen, C. Asplund, C.I. Smith, Microphthalmia, facial anomalies, microcephaly, thumb and hallux hypoplasia, and agammaglobulinemia, American journal of medical genetics, 101 (2001) 209-212.
[22] D. Çağdaş, T.T. Özgür, G.T. Asal, P. Revy, J.P. De Villartay, M. van der Burg, Ö. Sanal, I. Tezcan, Two SCID cases with Cernunnos-XLF deficiency successfully treated by hematopoietic stem cell transplantation, Pediatric transplantation, 16 (2012) E167-171.
[23] F.E. Cipe, C. Aydogmus, A. Babayigit Hocaoglu, M. Kilic, G.D. Kaya, E. Yilmaz Gulec, Cernunnos/XLF Deficiency: A Syndromic Primary Immunodeficiency, Case reports in pediatrics, 2014 (2014) 614238.
[24] T. Patiroglu, H.H. Akar, M. van der Burg, O. Kontas, A case of XLF deficiency presented with diffuse large B cell lymphoma in the brain, Clinical immunology (Orlando, Fla.), 161 (2015) 394-395.
[25] H.H. Akar, T. Patiroglu, M. Hershfield, M. van der Burg, Combined immunodeficiencies: twenty years experience from a single center in Turkey, Central-European journal of immunology, 41 (2016) 107-115.
[26] I.J. H, J. Rozmus, K. Schwarz, R.L. Warren, D. van Zessen, R.A. Holt, I. Pico-Knijnenburg, E. Simons, I. Jerchel, A. Wawer, M. Lorenz, T. Patıroğlu, H.H. Akar, R. Leite, N.S. Verkaik, A.P. Stubbs, D.C. van Gent, XLF deficiency results in reduced N-nucleotide addition during V(D)J recombination, 128 (2016) 650-659.
[27] J. Carrillo, O. Calvete, L. Pintado-Berninches, C. Manguan-García, J. Sevilla Navarro, E.G. Arias-Salgado, L. Sastre, G. Guenechea, E. López Granados, J.P. de Villartay, P. Revy, J. Benitez, R. Perona, Mutations in XLF/NHEJ1/Cernunnos gene results in downregulation of telomerase genes expression and telomere shortening, Hum Mol Genet, 26 (2017) 1900-1914.
[28] F. Sheikh, A. Hawwari, S. Alhissi, S. Al Gazlan, H. Al Dhekri, A.M. Rehan Khaliq, E. Borrero, L. El-Baik, R. Arnaout, H. Al-Mousa, A.M. Alazami, Loss of NHEJ1 Protein Due to a Novel Splice Site Mutation in a Family Presenting with Combined Immunodeficiency, Microcephaly, and Growth Retardation and Literature Review, Journal of clinical immunology, 37 (2017) 575-581.
[29] R. Yazdani, H. Abolhassani, J. Tafaroji, G. Azizi, A.A. Hamidieh, J. Chou, R.S. Geha, A. Aghamohammadi, Cernunnos deficiency associated with BCG adenitis and autoimmunity: First case from the national Iranian registry and review of the literature, Clinical immunology (Orlando, Fla.), 183 (2017) 201-206.
[30] L. Kager, R. Jimenez Heredia, T. Hirschmugl, J. Dmytrus, A. Krolo, H. Müller, C. Bock, P. Zeitlhofer, M. Dworzak, G. Mann, W. Holter, O. Haas, K. Boztug, Targeted mutation screening of 292 candidate genes in 38 children with inborn haematological cytopenias efficiently identifies novel disease-causing mutations, 182 (2018) 251-258.
[31] M.J. Recio, N. Dominguez-Pinilla, M.S. Perrig, C. Rodriguez Vigil-Iturrate, N. Salmón-Rodriguez, C. Martinez Faci, M.J. Castro-Panete, J. Blas-Espada, M. López-Nevado, R. Ruiz-Garcia, R. Chaparro-García, L.M. Allende, L.I. Gonzalez-Granado, Extreme Phenotypes With Identical Mutations: Two Patients With Same Non-sense NHEJ1 Homozygous Mutation, Frontiers in immunology, 9 (2018) 2959.

[32] H. Esmaeilzadeh, M.R. Bordbar, Z. Hojaji, P. Habibzadeh, D. Afshinfar, M. Miryounesi, An immunocompetent patient with a nonsense mutation in NHEJ1 gene, 20 (2019) 45.
[33] S. Firtina, Y. Yin Ng, O. Hatirnaz Ng, A. Kiykim, E. Aydiner, S. Nepesov, Y. Camcioglu, E.H. Sayar, I. Reisli, S.H. Torun, T. Cogurlu, D. Uygun, I.E. Simsek, A. Kaya, F. Cipe, D. Cagdas, E. Yucel, S. Cekic, V. Uygun, S. Baris, A. Ozen, U. Ozbek, M. Sayitoglu, Mutational landscape of severe combined immunodeficiency patients from Turkey, International journal of immunogenetics, 47 (2020) 529-538.
[34] R. Al-Marhoobi, M. Al-Musalhi, S.U. Naseem, Y. Wali, A. Alsayegh, S. Al-Tamemi, Combined Immunodeficiency, Hemolytic Anemia, and Growth Retardation Secondary to a Homozygous Mutation in the NHEJ1 Gene, Journal of pediatric hematology/oncology, 42 (2020) 333-335.
[35] A.K. Arunachalam, M. Maddali, F.N. Aboobacker, A. Korula, B. George, V. Mathews, E.S. Edison, Primary Immunodeficiencies in India: Molecular Diagnosis and the Role of Next-Generation Sequencing, 41 (2021) 393-413.
[36] M. Miano, A. Grossi, G. Dell'Orso, M. Lanciotti, F. Fioredda, E. Palmisani, Genetic screening of children with marrow failure. The role of primary Immunodeficiencies, 96 (2021) 1077-1086.
[37] P. Vignesh, A. Rawat, R. Kumrah, A. Singh, A. Gummadi, M. Sharma, A. Kaur, J. Nameirakpam, A. Jindal, D. Suri, A. Gupta, A. Khadwal, B. Saikia, R.W. Minz, K. Sharma, M. Desai, P. Taur, V. Gowri, A. Pandrowala, A. Dalvi, N. Jodhawat, P. Kambli, M.R. Madkaikar, S. Bhattad, S. Ramprakash, R. Cp, A. Jayaram, M. Sivasankaran, D. Munirathnam, S. Balaji, A. Rajendran, A. Aggarwal, K. Singh, F. Na, B. George, A. Mehta, H.P. Lashkari, R. Uppuluri, R. Raj, S. Bartakke, K. Gupta, S. Sreedharanunni, Y. Ogura, T. Kato, K. Imai, K.W. Chan, D. Leung, O. Ohara, S. Nonoyama, M. Hershfield, Y.L. Lau, S. Singh, Clinical, Immunological, and Molecular Features of Severe Combined Immune Deficiency: A Multi-Institutional Experience From India, Frontiers in immunology, 11 (2020) 619146.
[38] Y. Farsi, M. Hojabri, G. Eslamian, B.S. Shamsian, R. Mansour Ghanaie, M. Keramatipour, Z. Chavoshzadeh, S. Eskandarzadeh, A case of cernunnos immunodeficiency with a novel genetic mutation, Available at SSRN 3997299, (2021).
[39] M. Jamee, N. Khakbazan Fard, S. Fallah, Z. Golchehre, M. Fallahi, B.S. Shamsian, S. Sharafian, Z. Chavoshzadeh, Cernunnos defect in an Iranian patient with T(-) B(+) NK(+) severe combined immunodeficiency: A case report and review of the literature, Molecular genetics & genomic medicine, 10 (2022) e1990.
[40] S. Frizinsky, E. Rechavi, O. Barel, Y.N. Lee, A.J. Simon, A. Lev, T. Stauber, E. Adam, R. Somech, Novel NHEJ1 pathogenic variant linked to severe combined immunodeficiency, microcephaly, and abnormal T and B cell receptor repertoires, Frontiers in pediatrics, 10 (2022) 883173.
[41] B. Wolska-Kuśnierz, H. Gregorek, K. Chrzanowska, B. Piątosa, B. Pietrucha, E. Heropolitańska-Pliszka, M. Pac, M. Klaudel-Dreszler, L. Kostyuchenko, S. Pasic, Nijmegen breakage syndrome: clinical and immunological features, long-term outcome and treatment options–a retrospective analysis, Journal of clinical immunology, 35 (2015) 538-549.
[42] B.E. Hasbaoui, A. Elyajouri, R. Abilkassem, A. Agadr, Nijmegen breakage syndrome: case report and review of literature, The Pan African medical journal, 35 (2020) 85.
[43] S.O. Sharapova, E.I. Golovataya, E.V. Shepelevich, Y.E. Mareika, I.E. Guryanova, M.V. Stegantseva, O.V. Aleinikova, Nijmegen breakage syndrome in two half sibs with peripheral T-cell lymphoma and cortical T-cell acute lymphoid leukemia, Central-European journal of immunology, 45 (2020) 507-510.
[44] I. The, Nijmegen breakage syndrome. The International Nijmegen Breakage Syndrome Study Group, Archives of disease in childhood, 82 (2000) 400-406.
[45] M. Erdös, B. Tóth, I. Veres, M. Kiss, E. Remenyik, L. Maródi, Nijmegen breakage syndrome complicated with primary cutaneous tuberculosis, The Pediatric infectious disease journal, 30 (2011) 359-360.
[46] S. Kleier, M. Herrmann, B. Wittwer, R. Varon, A. Reis, J. Horst, Clinical presentation and mutation identification in the NBS1 gene in a boy with Nijmegen breakage syndrome, Clinical genetics, 57 (2000) 384-387.

[47] P. Maraschio, C. Danesino, A. Antoccia, R. Ricordy, C. Tanzarella, R. Varon, A. Reis, D. Besana, A. Guala, L. Tiepolo, A novel mutation and novel features in Nijmegen breakage syndrome, J Med Genet, 38 (2001) 113-117.
[48] C.A. Vogel, E.J. Stratman, S.J. Reck, J.J. Lund, Chronic Noninfectious Necrotizing Granulomas in a Child with Nijmegen Breakage Syndrome, Pediatric Dermatology, 27 (2010) 285-289.
[49] F.F.İ. Kardeş, Nijmegen-Breakage Syndrome; Two Siblings Presenting with Different Phenotypes.
[50] A. Filipiuk, A. Kozakiewicz, K. Kośmider, K. Karska, M. Lejman, J. Zawitkowska, Clinical manifestations, toxicities, and outcome of two children with Nijmegen breakage syndrome and lymphoid malignancies–case reports, Pediatria Polska-Polish Journal of Paediatrics, 97 (2022).
[51] T.I. Ben-Omran, K. Cerosaletti, P. Concannon, S. Weitzman, M.M. Nezarati, A patient with mutations in DNA Ligase IV: clinical features and overlap with Nijmegen breakage syndrome, American Journal of Medical Genetics Part A, 137 (2005) 283-287.
[52] N. Toita, N. Hatano, S. Ono, M. Yamada, R. Kobayashi, I. Kobayashi, N. Kawamura, M. Okano, A. Satoh, A. Nakagawa, Epstein–Barr virus-associated B-cell lymphoma in a patient with DNA ligase IV (LIG4) syndrome, American journal of medical genetics Part A, 143 (2007) 742-745.
[53] A.J. Jauch, O. Bignucolo, S. Seki, M. Ghraichy, O.M. Delmonte, V. von Niederhäusern, R. Higgins, A. Ghosh, M. Nishizawa, M. Tanaka, A. Baldrich, J. Köppen, J.R. Hirsiger, R. Hupfer, S. Ehl, A. Rensing-Ehl, H. Hopfer, S.S. Prince, S.R. Daley, F.A. Marquardsen, B.J. Meyer, M. Tamm, T.D. Daikeler, T. Diesch, T. Kühne, A. Helbling, C. Berkemeier, I. Heijnen, A.A. Navarini, J. Trück, J.P. de Villartay, A. Oxenius, C.T. Berger, C. Hess, L.D. Notarangelo, H. Yamamoto, M. Recher, Autoimmunity and immunodeficiency associated with monoallelic LIG4 mutations via haploinsufficiency, The Journal of allergy and clinical immunology, 152 (2023) 500-516.
[54] P.N. Plowman, B.A. Bridges, C.F. Arlett, A. Hinney, J.E. Kingston, An instance of clinical radiation morbidity and cellular radiosensitivity, not associated with ataxia-telangiectasia, The British journal of radiology, 63 (1990) 624-628.
[55] E. Riballo, S.E. Critchlow, S.H. Teo, A.J. Doherty, A. Priestley, B. Broughton, B. Kysela, H. Beamish, N. Plowman, C.F. Arlett, A.R. Lehmann, S.P. Jackson, P.A. Jeggo, Identification of a defect in DNA ligase IV in a radiosensitive leukaemia patient, Current biology : CB, 9 (1999) 699-702.
[56] A. Hayani, C.R. Suarez, Z. Molnar, M. LeBeau, J. Godwin, Acute myeloid leukaemia in a patient with Seckel syndrome, Journal of medical genetics, 31 (1994) 148-149.
[57] M. O'Driscoll, K.M. Cerosaletti, P.M. Girard, Y. Dai, M. Stumm, B. Kysela, B. Hirsch, A. Gennery, S.E. Palmer, J. Seidel, R.A. Gatti, R. Varon, M.A. Oettinger, H. Neitzel, P.A. Jeggo, P. Concannon, DNA ligase IV mutations identified in patients exhibiting developmental delay and immunodeficiency, Molecular cell, 8 (2001) 1175-1185.
[58] T.I. Ben-Omran, K. Cerosaletti, P. Concannon, S. Weitzman, M.M. Nezarati, A patient with mutations in DNA Ligase IV: clinical features and overlap with Nijmegen breakage syndrome, American journal of medical genetics. Part A, 137a (2005) 283-287.
[59] M. van der Burg, L.R. van Veelen, N.S. Verkaik, W.W. Wiegant, N.G. Hartwig, B.H. Barendregt, L. Brugmans, A. Raams, N.G. Jaspers, M.Z. Zdzienicka, J.J. van Dongen, D.C. van Gent, A new type of radiosensitive T-B-NK+ severe combined immunodeficiency caused by a LIG4 mutation, J Clin Invest, 116 (2006) 137-145.
[60] A. Enders, P. Fisch, K. Schwarz, U. Duffner, U. Pannicke, E. Nikolopoulos, A. Peters, M. Orlowska-Volk, D. Schindler, W. Friedrich, B. Selle, C. Niemeyer, S. Ehl, A severe form of human combined immunodeficiency due to mutations in DNA ligase IV, Journal of immunology (Baltimore, Md. : 1950), 176 (2006) 5060-5068.
[61] D. Buck, D. Moshous, R. de Chasseval, Y. Ma, F. le Deist, M. Cavazzana-Calvo, A. Fischer, J.L. Casanova, M.R. Lieber, J.P. de Villartay, Severe combined immunodeficiency and microcephaly in siblings with hypomorphic mutations in DNA ligase IV, European journal of immunology, 36 (2006) 224-235.

[62] M. Huang, G. Dong, DNA ligase IV dificiency with elevated serum IgG levels suspected to have myelodysplastic syndrome: a case report, 22 (2022) 588.
[63] E. Grunebaum, A. Bates, C.M. Roifman, Omenn syndrome is associated with mutations in DNA ligase IV, The Journal of allergy and clinical immunology, 122 (2008) 1219-1220.
[64] J.E. Murray, L.S. Bicknell, G. Yigit, A.L. Duker, M. van Kogelenberg, S. Haghayegh, D. Wieczorek, H. Kayserili, M.H. Albert, C.A. Wise, J. Brandon, T. Kleefstra, A. Warris, M. van der Flier, J.S. Bamforth, K. Doonanco, L. Adès, A. Ma, M. Field, D. Johnson, F. Shackley, H. Firth, C.G. Woods, P. Nürnberg, R.A. Gatti, M. Hurles, M.B. Bober, B. Wollnik, A.P. Jackson, Extreme growth failure is a common presentation of ligase IV deficiency, Human mutation, 35 (2014) 76-85.
[65] K. Felgentreff, S.N. Baxi, Y.N. Lee, K. Dobbs, L.A. Henderson, K. Csomos, E.N. Tsitsikov, M. Armanios, J.E. Walter, L.D. Notarangelo, Ligase-4 Deficiency Causes Distinctive Immune Abnormalities in Asymptomatic Individuals, Journal of clinical immunology, 36 (2016) 341-353.
[66] S.O. Sharapova, E.Y. Chang, I.E. Guryanova, I.V. Proleskovskaya, A.S. Fedorova, E.A. Rutskaya, O.V. Aleinikova, Next generation sequencing revealed DNA ligase IV deficiency in a "developmentally normal" patient with massive brain Epstein-Barr virus-positive diffuse large B-cell lymphoma, Clinical immunology (Orlando, Fla.), 163 (2016) 108-110.
[67] R. Dard, B. Herve, T. Leblanc, J.P. de Villartay, L. Collopy, T. Vulliami, S. Drunat, S. Gorde, A. Babik, P.F. Souchon, A. Agadr, R. Abilkassem, M. Elalloussi, A. Verloes, M. Doco-Fenzy, DNA ligase IV deficiency: Immunoglobulin class deficiency depends on the genotype, Pediatric allergy and immunology : official publication of the European Society of Pediatric Allergy and Immunology, 28 (2017) 298-303.
[68] A. Fadda, F. Butt, S. Tomei, S. Deola, B. Lo, A. Robay, A. Al-Shakaki, N. Al-Hajri, R. Crystal, M. Kambouris, E. Wang, F.M. Marincola, K.A. Fakhro, C. Cugno, Two hits in one: whole genome sequencing unveils LIG4 syndrome and urofacial syndrome in a case report of a child with complex phenotype, BMC medical genetics, 17 (2016) 84.
[69] C. Cifaldi, G. Angelino, M. Chiriaco, S. Di Cesare, A. Claps, J. Serafinelli, P. Rossi, A. Antoccia, G. Di Matteo, C. Cancrini, J.P. De Villartay, A. Finocchi, Late-onset combined immune deficiency due to LIGIV mutations in a 12-year-old patient, Pediatric allergy and immunology : official publication of the European Society of Pediatric Allergy and Immunology, 28 (2017) 203-206.
[70] A.T. Staines Boone, I.K. Chinn, C. Alaez-Versón, M.A. Yamazaki-Nakashimada, K. Carrillo-Sánchez, M.L.H. García-Cruz, M.C. Poli, M.E. González Serrano, E.A. Medina Torres, D. Muzquiz Zermeño, L.R. Forbes, F.J. Espinosa-Rosales, S.E. Espinosa-Padilla, J.S. Orange, S.O. Lugo Reyes, Failing to Make Ends Meet: The Broad Clinical Spectrum of DNA Ligase IV Deficiency. Case Series and Review of the Literature, Frontiers in pediatrics, 6 (2018) 426.
[71] M.Y. Zhang, S.B. Keel, T. Walsh, M.K. Lee, S. Gulsuner, A.C. Watts, C.C. Pritchard, S.J. Salipante, M.R. Jeng, I. Hofmann, D.A. Williams, M.D. Fleming, J.L. Abkowitz, M.C. King, A. Shimamura, Genomic analysis of bone marrow failure and myelodysplastic syndromes reveals phenotypic and diagnostic complexity, Haematologica, 100 (2015) 42-48.
[72] C.M. Bacon, S.J. Wilkinson, G.P. Spickett, D. Barge, H.H. Lucraft, G. Jackson, V. Rand, A.R. Gennery, Epstein-Barr virus-independent diffuse large B-cell lymphoma in DNA ligase 4 deficiency, The Journal of allergy and clinical immunology, 131 (2013) 1237-1239, 1239.e1231.
